# Integrating Eye Tracking in Acoustic Research: Methods for Sound Localization, Event Detection, Multimodal Sensing, and Perceptual Analysis

**DOI:** 10.3390/s26113603

**Published:** 2026-06-05

**Authors:** Giuseppe Ciaburro, Virginia Puyana-Romero

**Affiliations:** 1 School of Engineering and Informatics, Department of Engineering, Pegaso University, 80143 Naples, Italy; 2 Departamento de Ingeniería en Sonido y Acústica, Universidad de Las Américas, Quito 17513, Ecuador; virginia.puyana.romero@udla.edu.ec

**Keywords:** eye tracking, acoustic sensing, multimodal integration, sound localization, human perception

## Abstract

Recent advances in eye-tracking technologies have fostered growing interest in their integration with acoustic research for investigating auditory perception and human behavioral responses. This study presents a structured literature review of recent developments at the intersection of eye tracking and acoustics, with the aim of analyzing how eye-movement data can support the interpretation of auditory events, spatial listening behaviors, and multimodal human–environment interactions. The reviewed studies were organized into four main research areas focusing on the application of eye-tracking in acoustics: sound source localization and identification, sound event detection and classification, acoustic sensing and multimodal systems, and soundscape and perceptual acoustic studies. The analysis indicates that eye-movement patterns can provide useful indicators of auditory attention and perceptual processes, particularly when combined with complementary physiological, visual, and acoustic sensing modalities. Furthermore, recent methodological advances, including real-time processing, machine learning algorithms, and sensor fusion techniques, have contributed to improving the robustness and accuracy of multimodal data analysis. Nevertheless, the review also highlights several limitations in current research, such as the lack of standardized experimental protocols, inter-individual variability, and susceptibility to environmental noise and external interference. Finally, future research perspectives are discussed, emphasizing the development of standardized and adaptive multimodal frameworks for human behavior modeling and intelligent acoustic monitoring systems.

## 1. Introduction

Over the past few decades, the field of acoustics has gradually become more interdisciplinary, shifting from a relatively narrow physical and engineering discipline to one characterized by a significant involvement of issues relating to the human perception of sounds. It is worth noting that the importance of understanding the mechanisms governing human perception, interpretation, and response to acoustic stimuli is now relevant both in classical acoustic applications and in such emerging disciplines as human–computer interaction, virtual and augmented reality, and intelligent surveillance [1,2]. In this regard, among various new technologies currently being researched by the scientific community, there is one that deserves special attention—namely, eye tracking.

Eye tracking refers to recording and analyzing eye movements [3]. Thanks to the analysis of fixations, saccades, gaze durations, and eye trajectories, one can get valuable in-formation about how visual attention is distributed both in space and in time [4]. Even though this technology has been extensively used in many fields of science, ranging from cognitive psychology to marketing and neurosciences, only recently researchers became increasingly interested in applying eye tracking in acoustics [5]. The reason for that is the recognition of the fact that auditory perception is largely influenced by the integration of input coming from different sensory systems, most notably vision.

The application of eye tracking in acoustics is very diverse. Among the problems where eye movement measurements have proven to be especially useful, one can mention source localization and identification [6]. There are plenty of studies showing that eye movements can shed light on how people focus their attention on a certain acoustic source when there are multiple stimuli present in the environment [7]. Audio–visual interactions are of particular interest in this respect since the presence of congruent or incongruent visual information affects how subjects localize the sound [8]. As a result, the study of multisensory integration based on the analysis of eye movements becomes highly informative [9].

A second research area that may benefit from eye tracking technology is the detection and classification of sound events. By combining eye tracking data with methods for acoustic signal processing and machine learning, researchers could develop novel approaches for detecting sounds [10]. Specifically, the analysis of eye patterns could prove to be valuable information for training models that classify sound events. As eye movements give information about the attention level of the person and the perceived relevance of the acoustic stimulus, these data could improve the performance of classification algorithms [11]. Such applications have many practical implications, such as surveillance systems that detect threatening sounds, medical tools for diagnosing conditions based on sounds, and devices for environmental monitoring and security [12].

Simultaneously, eye tracking has also been adopted for designing multimodal acoustic sensing systems. These devices integrate data collected through acoustic sensors with visual and behavioral information to produce a comprehensive representation of the surrounding environment [13]. The integration of eye data allows the design of systems that adapt to the specific behavior and preferences of users [14]. From this perspective, the use of eye tracking in acoustic sensing systems can be considered an aspect of the wider field of intelligent systems and artificial intelligence, which relies heavily on data fusion techniques.

Finally, a third research area involving the combination of eye tracking and acoustics is concerned with soundscape and perceptual acoustics studies. In this case, researchers are interested in investigating how people perceive soundscapes considering not only the physical characteristics of sounds, but also cultural and contextual dimensions [15]. Eye tracking is an important tool for conducting soundscape studies since it makes it possible to quantify visual attention across an environment and examine how this information contributes to the overall perception of the soundscape [16]. Thus, by combining eye tracking and acoustics, researchers can determine the effects of visual and contextual elements on perception. For instance, eye tracking can help researchers explore how people perceive urban noises in different situations. It could be interesting to assess the role of visual stimuli, both natural and man-made, on the evaluation of soundscapes. Furthermore, researchers could study how the interactions with virtual environments modify people’s perceptions.

The integration of eye tracking and acoustic analysis can be interpreted within the broader framework of multisensory perception, where auditory and visual information are dynamically combined to support human interaction with the surrounding environment. According to multisensory integration theories, sensory modalities do not operate independently; rather, the brain continuously combines visual, auditory, and physiological cues to enhance perceptual accuracy and spatial awareness [17]. In this context, eye movements represent an important behavioral indicator for investigating attentional mechanisms, perceptual responses, and cognitive processing associated with auditory stimuli.

Auditory attention refers to the cognitive ability to selectively focus on relevant acoustic information while filtering competing stimuli from the surrounding environment [18]. Eye tracking measurements, including fixation patterns, saccades, blink rate, and pupil dilation, have increasingly been employed as indirect indicators of attentional allocation and listening effort, particularly in complex acoustic scenarios. At the same time, multimodal integration approaches combine eye tracking data with acoustic, physiological, and contextual measurements to improve the interpretation of human behavior and environmental perception.

Furthermore, recent advances in intelligent sensing systems, machine learning, and real-time data processing have expanded the applicability of eye–acoustic methodologies in domains such as human–computer interaction, immersive virtual environments, robotics, healthcare, and soundscape assessment. Rather than representing a complete paradigm shift, these developments indicate a progressive transition toward more adaptive and human-centered multimodal sensing frameworks capable of capturing the complexity of perceptual and cognitive processes.

Given all these aspects, it becomes evident that the adoption of eye tracking technology in acoustics can be considered both an important methodology improvement and a revolution in the research paradigm since it enables studying the interaction between the individual and the sound environment from the perspective of a subjective and user-centric approach that is required for various modern applications. Nonetheless, there are still many open questions in this research area, including the need for standardization of methodologies for acquisition and analysis of eye tracking data, its effective integration with other types of information, and, finally, the deep study of mechanisms underlying correlations between visual and acoustic signals.

In this regard, this article aims at providing a systematic and critical review of state-of-the-art research concerning the use of eye tracking in the scope of acoustics. The analysis will focus on the following main topics, such as sound source localization and identification, acoustic events detection and classification, multisensory systems using the combination of ocular and acoustic data, and soundscape and perceptual acoustics. In this way, it is expected to identify the main methods used in studies, the results obtained, and further directions of research in this fast-growing area.

The proposed review should serve as a means for bridging the gap between engineering solutions and perceptual studies in order to stimulate more interdisciplinary cooperation and the development of new technologies. Eye tracking, in this case, will enable researchers to gain deeper insights into the nature of relationships between the individual and the soundscape.

## 2. Foundations and Technological Evolution of Eye Tracking Systems

Eye tracking is a sophisticated technology, which can be used to measure and analysis eye movement and to get knowledge about attentional, cognitive, and perceptual process-es of the observer. Its main idea lies in the fact that eye movement is not random but corresponds to visual information processing and decision making. Therefore, it is possible to analyze indirect data about a person’s mental processes during communication with the surrounding world using eye tracking. It is a unique technique that helps study visual attention, human–computer interaction, and visual perception providing quantitative measurements of such phenomena that have never been studied before quantitatively.

**Figure 1:**
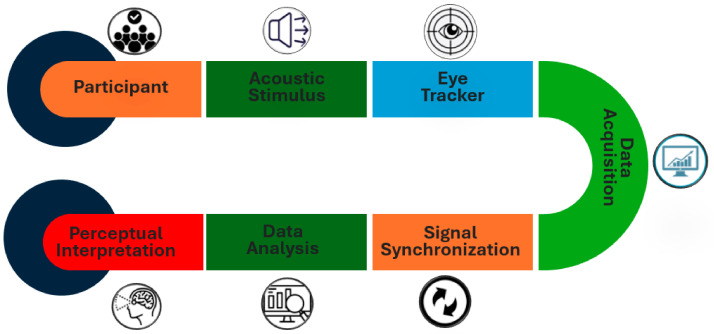
Flowchart illustrating the general workflow of eye-tracking integration in acoustic research. The path flow first obeys the sequence: Participant (P) → Acoustic Stimulus (AS) → Eye Tracker (ET) → Data Acquisition (DA). The first part of the process thus involves selecting a subject (P), who perceives an external sound (AS), during which subject’s eyes movements are recorder (ET), followed by storing the information in a database (DA). The second part of the flow consists of synchronizing the signal (Signal Synchronization), followed by a numerical analysis of the information (Data Analysis), and finally leading to the interpretation of the participant perception information.

Figure 1 illustrates the general methodological workflow adopted in eye-tracking acoustic studies, highlighting the sequential process from acoustic stimulus exposure and gaze recording to synchronized data analysis and perceptual interpretation. Participants are typically seated comfortably in a controlled laboratory environment to minimize movement artifacts and external disturbances. However, eye-tracking measurements can also be conducted in outdoor or real-world environments, particularly in soundscape studies, urban acoustic investigations, and ecological validity assessments. The choice of environment depends on the research objectives, the required level of experimental control, and the characteristics of the acoustic stimuli under investigation.

Signal synchronization (Figure 1) is a critical step in multimodal acoustic experiments involving eye tracking. The procedure consists of temporally aligning eye-tracking recordings with acoustic stimuli and, when available, additional physiological or environmental measurements. Synchronization ensures that gaze behavior can be accurately associated with specific sound events, enabling reliable interpretation of participants’ perceptual and attentional responses. Depending on the experimental setup, synchronization may be achieved through hardware triggers, shared timestamps, synchronization markers, or post-processing alignment techniques.

Technically, the main technique used to realize eye tracking nowadays is the video-oculography (VOG), which uses a video camera and an infrared illumination source to locate pupil position and reflection of light from the cornea. Analysis of the data obtained using these methods makes it possible to determine with a very high accuracy point of fixation of the gaze in the space. More precisely, geometric model of the human eye and calibration algorithms make it possible to map ocular coordinates to a two- or three-dimensional space [19]. In addition to VOG, there are also alternative techniques, such as electro-oculography (EOG), which measures the differences in electrical potential generated by eye movements. Although these are generally less precise, they are useful in specific contexts, such as clinical settings or in conditions of poor visibility.

The performance of eye tracking devices has improved considerably in recent times, with increased precision in spatial terms (below 0.5° visual angle) and higher temporal resolution, enabling the use of sampling rates above 1000 Hz in state-of-the-art equipment. This has been achieved through the availability of new optical sensors, advancements in imaging technology, and the application of artificial intelligence algorithms for identifying the relevant features of the eyes [20]. Moreover, today’s eye trackers have become increasingly resilient to different environmental conditions, such as lighting changes, head movements, or even glasses, expanding their scope of application.

There are various types of eye tracking devices, which include remote eye trackers (screen-based systems), wearable eye trackers, and eye trackers embedded within immersive environments. Remote eye trackers are typically laboratory-based systems that detect eye movements with very high precision but do not involve any direct physical interaction with the subject. However, their use is limited to relatively stable positions of the head. The other type is wearable eye trackers, such as eye tracking glasses, which provide greater flexibility in detecting eye movements in dynamic environments. These systems are particularly useful for ecological studies and for industrial, ergonomic, and behavioral applications. Finally, the integration of eye tracking into virtual reality (VR) and augmented reality (AR) platforms has opened new perspectives for analyzing human behavior in immersive environments, where it is possible to precisely control the presented stimuli while maintaining a high degree of realism [21].

With regard to the parameters studied, eye tracking makes it possible to assess different aspects of oculomotor activity, which may include fixations, saccades, smooth pursuits, and pupil dilation. The process of fixation implies periods when the eyes are directed at a certain location and is related to visual information processing. Saccades are fast ballistics when the eyes move to a particular point within the visual scene [22]. Smooth pursuit makes it possible for the eyes to follow an object in motion, while pupil dilation can be related to different cognitive and emotional processes like mental workload and interest level, for example. Analysis of all these eye movement-related parameters can help develop models of visual attention and explore more complicated phenomena, including attention selection and decision-making.

**Figure 2:**
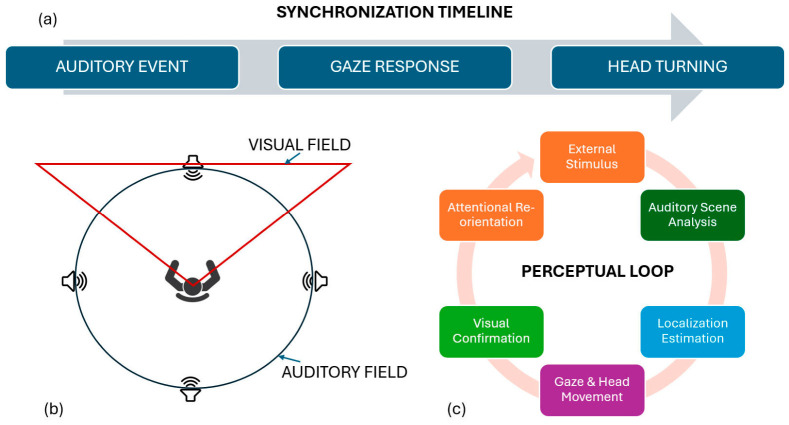
Cognitive and behavioral architecture of audio–visual spatial synchronization. (**a**) Synchronization timeline illustrating the sequential latency of motor responses following an acoustic event. The process begins with the onset of an Auditory Event, followed by an initial Gaze Response, and culminates in Head Turning, which serves to stabilize the visual field and refine spatial orientation. (**b**) Spatial field comparison showing the asymmetry between the omnidirectional 360° Auditory Field and the restricted, forward-facing Visual Field (represented by a triangular sector). Four sound source icons indicate potential peripheral or eccentric acoustic stimuli relative to the participant. (**c**) Perceptual loop describing the cyclical six-stage audio–visual processing model. The loop begins with the reception of an External Stimulus and proceeds through Auditory Scene Analysis and Localization Estimation, followed by motor responses (Gaze and Head Movement), and concludes with sensory validation stages (Visual Confirmation) and Attentional Re-orientation, which reset the system for subsequent stimulus processing.

To contextualize the behavioral and cognitive mechanisms of spatial orientation, Figure 2 illustrates the temporal and conceptual framework of audio–visual integration during a localization task. The process operates across both a linear timeline and a continuous feedback cycle, mapping how a subject interacts with their omnidirectional acoustic environment relative to their restricted visual field. As shown in Figure 2b, the auditory field encompasses a full 360° around the subject, whereas the visual field is restricted to a forward-facing wedge (indicated by the red boundaries). When an acoustic event occurs outside of this immediate visual field, the central nervous system must orchestrate a coordinated behavioral response to bring the source into focus. This response is governed by two complementary structures:

- Synchronization Timeline (Figure 2a): A sequential, feedforward progression of physical actions. It begins with the onset of the Auditory Event, followed rapidly by a reflexive Gaze Response (eye movement), and culminates in a compensatory Head Turning maneuver to align the head with the target.
- Perceptual Loop (Figure 2c): A continuous, cognitive–behavioral feedback loop that processes the event. An External Stimulus triggers an Auditory Scene Analysis, allowing the brain to compute a Localization Estimation. This estimation drives the physical Gaze & Head Movement, leading to a Visual Confirmation once the object enters the visual field. Finally, Attentional Re-orientation resets the system, preparing it for subsequent stimuli. Together, these mechanisms highlight how temporal motor synchronization serves the broader cognitive objective of sensory feedback and environmental mapping.

In Figure 2c, “Localization Estimation” refers to the perceptual and computational process of determining the spatial position of a sound source, i.e., identifying its perceived direction and distance in the acoustic scene. “Visual Confirmation” represents the subsequent gaze validation stage, in which the observer’s eye movements are directed toward the estimated sound source location to verify or refine the auditory-based spatial estimate. This step is necessary to integrate auditory and visual cues and reduce ambiguity in source identification. “Attentional Reorientation” describes the cognitive and perceptual reset of visual attention after confirmation, enabling the subject to disengage from the current target and become receptive to new acoustic events. This mechanism is required to allow the cyclic processing of multiple sound events within dynamic acoustic environments, ensuring continuous updating of auditory–visual attention loops.

An important step during eye tracking procedure is calibration, when users should fixate their eyes on predetermined spatial locations so that their position in space is calculated with regard to the location of the computer screen or any environment [23]. The quality of the calibration directly impacts the accuracy of the collected data and represents a major challenge in dynamic application contexts or with non-collaborative users, such as children or clinical patients.

State-of-the-art research in the field of eye tracking technologies demonstrates increasing trends toward combining with artificial intelligence and machine learning. Modern algorithms, apart from enhancing gaze estimation, allow automatically splitting input sequences of data into different events (e.g., fixations and saccades) and recognizing sophisticated behavior patterns. In addition, deep learning solutions are gaining popularity as effective means of predicting the user’s behavior in terms of visual perception [24].

Another area in which significant progress can be observed is multimodal eye tracking analysis, which integrates information received via eye tracking with additional cues (e.g., EEG/ECG/GSR data, behavioral or environmental information). This approach provides researchers with richer data about the user’s current state, making it easier to analyze various cognitive processes taking place [25]. Multimodal solutions have become widely used in adaptive interfaces and other intelligent solutions, which can modify their behavior based on the information collected about the user’s current state.

Yet another relevant advance involves miniaturization and cost reductions, allowing the increasing spread of eye tracking systems outside research laboratories. Portable eye tracking equipment, capable of being incorporated into devices such as smartphones, virtual reality headsets or car dashboards, makes it possible to employ this technique more widely. For example, in the industry, it may be applied in order to enhance efficiency in production processes and to increase work safety; in marketing—to observe consumer behavior;

In training, it has been used to assess the efficacy of instructional material [26]. Eye tracking has become increasingly important as a diagnostic non-invasive method in clinical and neuroscience applications. This technique allows us to investigate neurological and psycho-pathological conditions, including Parkinson’s disease, autism, and attention-deficit disorders, and provides objective criteria of cognitive activity. In addition, this methodology has been successfully employed in rehabilitation and helping people with motor impairments using communication tools that are based solely on eye movements [27].

There are several unresolved problems in this field, including noise management, standardization of methods and equipment, comparison among different types of equipment, and creation of common metrics for eye data analysis. Also, ethical questions concerning privacy issues and the use of biometric data are gaining more relevance in connection with the use of these techniques on a commercial scale [28].

Thanks to the technological progression of eye tracking, the technique has become a flexible and scalable instrument that is increasingly embedded in more complex systems. Eye tracking’s capacity to deliver accurate and immediate data regarding cognitive and perceptual processes makes it an essential component of many applications [29]. Technological advancement becomes the starting point for the realization of the latest and most revolutionary applications, including the one based on acoustics, which combines visual and audio information [30].

Recent years have witnessed significant advancements in eye-tracking technologies, greatly expanding their applicability in acoustic research and multimodal perception studies. Initially developed primarily for psychological and usability investigations, modern eye-tracking systems have evolved into highly accurate, portable, and real-time sensing platforms capable of supporting complex analyses in dynamic environments. These developments have substantially improved the capability of researchers to investigate auditory perception, sound localization, attention mechanisms, and human interaction with acoustic environments.

One of the most important technological improvements concerns the increase in spatial and temporal resolution. Contemporary eye trackers can achieve sampling frequencies exceeding 1000 Hz with high gaze accuracy, allowing researchers to capture rapid eye movements such as microsaccades, fixations, and smooth pursuit behaviors with unprecedented precision. This enhanced performance is particularly relevant in acoustic studies involving rapid auditory events, spatial sound localization, or dynamic audiovisual stimuli, where precise synchronization between auditory signals and gaze behavior is essential. Higher temporal resolution also enables more reliable analyses of cognitive load and attentional shifts during listening tasks.

Another major development is the miniaturization and portability of eye-tracking devices. Traditional laboratory-based systems required controlled environments and fixed head positions, limiting ecological validity. In contrast, recent wearable eye trackers, including lightweight glasses-based systems, permit data acquisition in real-world conditions and immersive environments. This transition has facilitated the investigation of auditory perception in everyday scenarios such as urban soundscapes, classrooms, industrial environments, driving simulations, and virtual reality applications. Consequently, researchers can now analyze human responses to acoustic stimuli under more realistic behavioral and environmental conditions.

**Figure 3:**
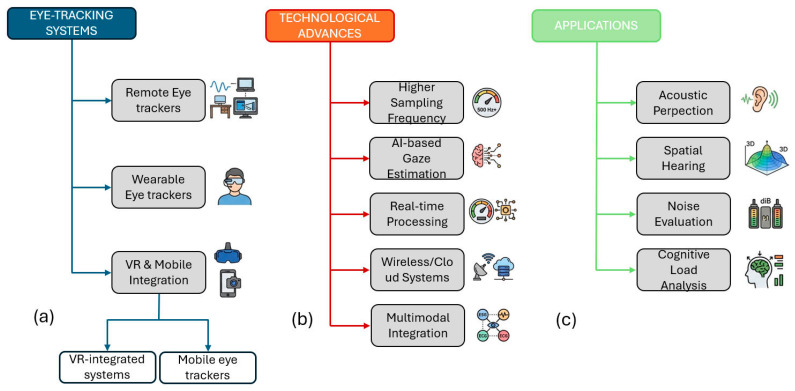
Taxonomic framework of eye-tracking methodologies, enabling technologies, and psychoacoustic applications: (**a**) Eye-Tracking Systems classify the available hardware configurations based on their operational context. This includes stationary systems (remote eye trackers) designed for controlled laboratory use, mobile systems (wearable eye trackers) suitable for naturalistic and field measurements, and immersive or portable configurations such as VR-integrated systems and mobile eye trackers, which enable gaze monitoring within interactive or simulated environments. (**b**) Technological Advances summarize the key developments driving the evolution of eye-tracking systems. These include high-frequency sampling rates for improved temporal resolution, artificial intelligence methods for predictive gaze estimation, real-time edge computing for on-device processing, cloud-based connectivity for large-scale data management, and multimodal sensor fusion approaches combining eye-tracking with physiological signals such as EEG and ECG. (**c**) Applications describe the main psychoacoustic and audiological domains supported by these technologies. These include the analysis of auditory-driven visual attention, spatial sound localization behavior, evaluation of environmental noise tolerance, and physiological assessment of cognitive and perceptual listening load in complex acoustic environments.

To understand the methodological and technological landscape underpinning contemporary gaze-tracking research in audiology and spatial audio, Figure 3 maps out a taxonomic overview of eye-tracking systems, their driving technological breakthroughs, and their primary research applications. This structural taxonomy underscores how evolution in hardware and computational power has directly expanded the horizons of auditory behavior analysis.

The architecture of this framework is divided into three interconnected domains:

- Eye-Tracking Systems: This domain categorizes the physical modalities of data collection. It differentiates between traditional desktop-bound Remote Eye trackers, head-mounted Wearable Eye trackers (such as specialized glasses), and the emerging frontier of VR & Mobile Integration, which is further bifurcated into fully immersive virtual reality environments and highly portable mobile tracking configurations.
- Technological Advances: This pillar highlights the computational and engineering milestones that have transformed eye-tracking from a laboratory novelty into a robust scientific instrument. Key innovations include Higher Sampling Frequencies (e.g., 500 Hz for capturing micro-saccades), AI-based Gaze Estimation algorithms, low-latency Real-time Processing, untethered Wireless/Cloud Systems, and Multimodal Integration frameworks that allow gaze data to be synchronously paired with physiological signals like EEG and ECG.
- Applications: The final domain bridges these hardware and software advancements with practical research in psychoacoustics. By leveraging highly accurate gaze metrics, researchers can assess foundational Acoustic Perception and Spatial Hearing dynamics (such as the localization behaviors explored in Figure 2). Furthermore, these systems facilitate real-world Noise Evaluation protocols and sophisticated Cognitive Load Analysis, evaluating the mental effort required to listen in challenging acoustic environments.

By mapping hardware modalities to specific technological drivers, this taxonomy demonstrates how modern eye-tracking paradigms enable deep, non-invasive insights into human auditory processing.

The integration of eye tracking with artificial intelligence and advanced computer vision techniques has further enhanced the analytical potential of these systems. Machine learning algorithms are increasingly employed for automated gaze classification, fixation detection, blink analysis, and behavioral prediction. These approaches improve the robustness of data interpretation while reducing the need for extensive manual processing. In acoustic research, AI-assisted eye-tracking analysis can support the identification of perceptual patterns related to sound source localization, speech intelligibility, auditory attention, and emotional responses to noise exposure.

Recent developments have also promoted the emergence of multimodal sensing frameworks in which eye tracking is combined with other physiological and environmental sensing technologies. Eye trackers are now frequently integrated with electroencephalography (EEG), electromyography (EMG), motion capture systems, biosensors, and acoustic measurement devices. Such multimodal approaches provide a more comprehensive understanding of human perception by simultaneously capturing visual attention, neural activity, body motion, and auditory responses. In particular, the combination of eye tracking and immersive virtual or augmented reality systems has become increasingly important for studying spatial hearing and interactive sound environments.

Cloud computing and wireless communication technologies have additionally contributed to the evolution of eye-tracking platforms. Modern systems increasingly support real-time data streaming, remote experimentation, and online collaborative analysis. These capabilities are especially valuable for large-scale perceptual studies and human-centered acoustic research involving geographically distributed participants.

Overall, the recent evolution of eye-tracking technologies has significantly broadened the methodological possibilities available in acoustic research. Improvements in accuracy, portability, multimodal integration, and intelligent data analysis have transformed eye tracking from a supplementary observational tool into a central methodology for investigating auditory perception and human interaction with complex acoustic environments.

The integration of eye-tracking technologies with acoustic sensing systems has progressively emerged as a powerful framework for modeling human behavior in complex auditory environments. This eye–acoustic coupling enables a multimodal representation of perceptual and cognitive processes, providing a more comprehensive understanding of how humans interact with, interpret, and respond to soundscapes. In particular, the simultaneous analysis of gaze behavior and acoustic stimuli allows researchers to move beyond traditional single-modality approaches, offering new insights into auditory attention, environmental awareness, and perceptual decision-making.

From a behavioral modeling perspective, eye movements represent a direct and high-resolution proxy of visual attention, which is strongly interconnected with auditory processing. Fixations, saccadic movements, and gaze transitions can reveal how individuals allocate attentional resources when exposed to competing or spatially distributed sound sources. For instance, in multi-source acoustic environments, gaze direction often aligns with perceived auditory salience, reflecting an implicit coupling between auditory localization and visual orientation mechanisms. This relationship becomes particularly relevant in scenarios involving auditory scene analysis, where the brain must segregate and prioritize relevant sound sources from background noise.

When integrated with acoustic measurements—such as sound pressure levels, spectral features, spatial cues, and temporal variations—eye-tracking data enables the construction of robust multimodal datasets for behavioral interpretation. These datasets facilitate the development of computational models that link environmental acoustic characteristics to observable human responses. In this context, eye–acoustic systems support the quantification of auditory attention shifts, enabling the identification of patterns in how users respond to changes in sound intensity, directionality, and complexity.

Recent advances in data-driven modeling approaches, including machine learning and probabilistic inference techniques, have further expanded the potential of eye–acoustic integration. These methods allow for the prediction of behavioral outcomes such as sound source localization accuracy, attentional focus distribution, and perceptual decision latency. In particular, supervised learning models can be trained to associate specific gaze patterns with corresponding acoustic conditions, while unsupervised approaches can uncover latent structures in multimodal behavioral data without predefined labels. As a result, eye–acoustic systems are increasingly being used to develop predictive models of human auditory behavior that are both adaptive and context sensitive.

Moreover, the incorporation of eye-tracking data into acoustic behavior modeling provides valuable indicators of cognitive load, situational awareness, and emotional response. Changes in fixation duration, pupil dilation, and gaze dispersion have been shown to correlate with increased cognitive effort during complex auditory tasks, such as speech-in-noise perception or navigation in dynamic acoustic environments. These metrics offer a non-invasive means of assessing mental workload and perceptual difficulty, which are critical parameters in human-centered acoustic design and evaluation.

Ecological validity is another important aspect of eye–acoustic behavior modeling. Wearable eye-tracking systems, combined with spatial audio reproduction technologies and virtual or augmented reality environments, enable the study of human behavior in realistic yet controllable settings. This allows researchers to replicate real-world auditory scenarios—such as urban environments, transportation hubs, or industrial spaces—while maintaining experimental precision. In these conditions, behavioral responses can be analyzed in relation to both visual and auditory contextual cues, leading to more generalizable findings.

Overall, eye–acoustic systems represent a significant advancement in human behavior modeling within acoustic research. By integrating visual attention metrics with detailed acoustic descriptors, these systems provide a multidimensional framework for understanding perception, cognition, and action in complex sound environments. This integrated approach not only enhances the interpretability of human responses but also supports the development of intelligent systems for environmental monitoring, adaptive sound design, and human–machine interaction in acoustically rich settings.

## 3. Materials and Methods

**Figure 4:**
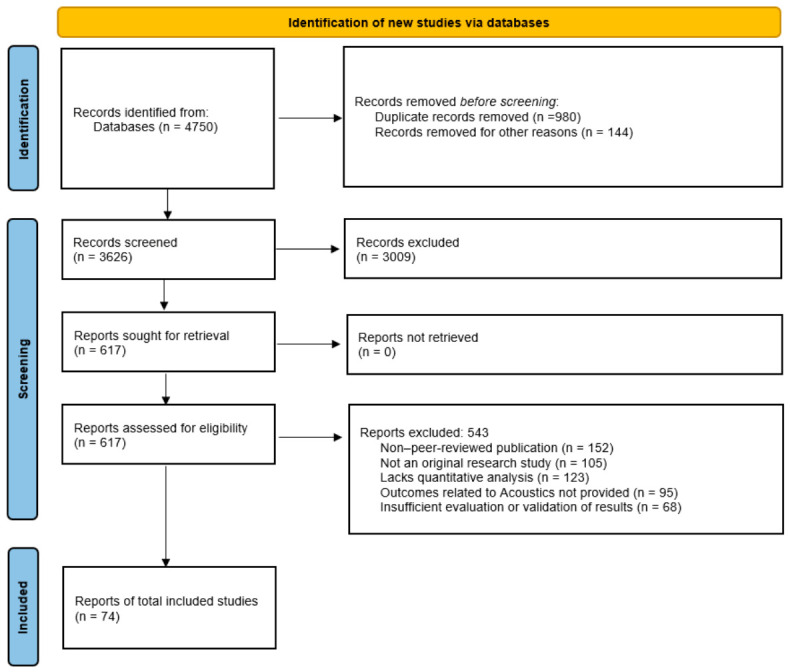
PRISMA 2020 flow diagram illustrating the study selection process. The numbers reported in each stage refer exclusively to the subset of records included in this systematic review after application of the predefined inclusion and exclusion criteria. The initial identification phase involved a larger pool of records, which were subsequently screened, assessed for eligibility, and refined to obtain the final dataset used in this study. Detailed information on the selection criteria and screening procedure is provided in the main text (see followings pharagraphs).

This systematic review is constructed according to a well-organized and strict methodology-based approach following the PRISMA (Preferred Reporting Items for Systematic Reviews and Meta-Analyses) recommendations that are widely used internationally as an example of transparency, reproducibility, and scientific rigor in literature reviews [31,32,33] (Figure 4). With the use of the above-mentioned methodology, the possibility of developing a consistent structure of all research steps from formulating its goals up to drawing conclusions from the results obtained was ensured.

The methodology-based process included several consecutive stages. First, the research question was defined using the PI-COS (Population, Intervention, Comparison, Outcomes, Study Design) framework, appropriately adapted to the interdisciplinary context of acoustics and eye tracking [34,35]. Specifically, the “population” was identified as the set of scientific studies analyzing the use of eye tracking in acoustic research; the “intervention” concerned the eye tracking technologies, experimental methodologies, and computational models used; the “comparison” was defined in relation to traditional acoustic techniques or approaches lacking visual integration; finally, the “outcomes” were identified as system performance, the ability to interpret perceptual phenomena, and the improvements brought about by multimodal integration.

Based on this framework, four research questions were formulated, consistent with the main thematic directions of this review. They aim to clarify:

- RQ1: How is eye tracking used to support sound source localization and identification in acoustic research?
- RQ2: What methods and models integrate eye tracking data for sound event detection and classification tasks?
- RQ3: How does eye tracking contribute to the development of multimodal acoustic sensing systems and human–machine interaction frameworks?
- RQ4: What insights does eye tracking provide in the assessment of soundscapes and human perceptual responses to acoustic environments?

These guiding questions shaped all aspects of the literature review process. The first stage of bibliographic searches was held from September 2025 to April 2026, during which a set of scientific databases such as Scopus, Web of Science, PubMed, IEEE Xplore, and ScienceDirect, as well as Google Scholar, was used to conduct a search. The selected time span focused primarily on recent contributions published in the last decade, reflecting the rapid evolution of eye tracking technologies, wearable sensing systems, machine learning methods, and immersive multimodal environments. Earlier foundational studies were included when considered particularly relevant to the theoretical framework of the review.

This selection of sources stems from a necessity to thoroughly cover both engineering and technological as well as perceptual and cognitive literature on the topic. The search was designed through the iterative use of keywords (“eye tracking,” “auditory attention, “ “acoustic perception,” “sound localization,” “multimodal sensing,” “sound event detection”, “sensor fusion”) along with the Boolean operator, synonymy, and term variations to widen the scope and minimize possible biases.

To reduce potential selection bias, the screening process was independently performed through multiple evaluation stages involving title, abstract, and full-text analysis. Discrepancies in study inclusion were discussed collectively until consensus was reached. The quality assessment considered methodological clarity, reproducibility of experimental procedures, dataset transparency, integration of multimodal sensing modalities, and robustness of data analysis techniques.

Due to the substantial heterogeneity of the reviewed studies in terms of experimental protocols, sensing technologies, participant characteristics, and evaluation metrics, a formal meta-analysis was not considered appropriate. Instead, a structured comparative synthesis was adopted to qualitatively analyze methodological approaches, application domains, sensing configurations, and reported performance indicators across the selected studies.

Finally, strict inclusion and exclusion criteria were applied to the selection of articles in order to ensure objectivity [36,37]. Peer-reviewed, full-text articles in English dealing with the topic of eye tracking within the acoustic environment were included. Experimental and theoretical contributions presenting quantitative results were also considered. Redundant studies, those that lacked peer-review, narrative reviews, editorials, studies that did not meet methodological quality standards or had no relevance to the study goals were also excluded from analysis. Special care was taken in the exclusion of studies marked by lack of transparency, inappropriate samples or results that could not be reproduced.

The study selection process involved three main steps: In the first phase, all identified records were imported into bibliographic management software, allowing for the automatic elimination of duplicates. Subsequently, two independent reviewers performed a preliminary screening based on titles and abstracts, excluding manifestly irrelevant contributions. In the final phase, eligible articles were fully analyzed to verify their compliance with the established criteria. Any disagreements between reviewers were resolved through collegial discussion or, where necessary, through the involvement of a third reviewer. The entire process was documented using the PRISMA flowchart [38], which detailed the number of studies identified, selected, and excluded at each stage.

Once the final corpus of studies was defined, the data extraction phase began using a specially designed standardized form. This tool allowed for the systematic collection of information regarding bibliographical aspects (authors, year, publication location), methodological characteristics (experimental design, sample type, instruments used), main results, and limitations highlighted by the authors. Furthermore, specific details were collected regarding the datasets used, the ocular and acoustic signal preprocessing techniques, the analysis algorithms, and the evaluation metrics adopted. This approach ensured consistency and accuracy in the synthesis phase.

To assess the quality of the included studies, a qualitative analysis was conducted based on criteria such as methodological rigor, clarity in data description, consistency of results, and replicability of experiments. The risk of bias was assessed by considering factors such as selective reporting, potential discrepancies between methodology and results, and the presence of publication bias. However, formal quantitative tools for measuring bias were not applied, as the heterogeneity of the analyzed studies precluded it.

The synthesis of the results was conducted primarily through a qualitative and narrative approach, organizing the contributions according to the four identified thematic areas. This choice was motivated by the high variability in experimental designs, application contexts, and evaluation metrics, which made direct quantitative comparisons difficult. However, where possible, recurring trends, points of convergence and divergence between studies, as well as gaps in the literature, were highlighted. Moreover, the review took into consideration cross-cutting issues, like the application setting (real, simulated, virtual environment), the technology used, and the degree of sensory modality integration.

Lastly, special focus was put on examining the heterogeneous nature of the reviewed literature, which has been studied in view of the datasets involved, the experimental setup, and the goals pursued by each study. The heterogeneity aspect has been not only taken into account as a limiting factor, but also seen as a sign of the multidimensionality and richness of the scientific domain, emphasizing the highly interdisciplinarity of the eye tracking–acoustic interaction field. All in all, the chosen methodology has provided us with good grounds for an informed and thorough synthesis of the state of the art in question.

## 4. Results

The following paragraphs present a critical and structured synthesis of the results yielded by the systematic literature review concerning the use of eye tracking in acoustics research. As per the chosen methodology, the selected papers were grouped according to four thematic macro-categories that have been established a priori according to the research questions proposed (RQ1-4). In this way, it becomes possible to clearly identify how eye tracking techniques have been applied in the acoustics context, highlighting convergences and divergences both in methodological terms and in terms of topics studied and investigated. For each thematic macro-category, a descriptive discussion of the studies involved is provided in order to pinpoint recurrent experimental techniques used, technologies applied, metrics considered for measuring eye movements, and outcomes achieved. Of course, the importance of multimodal processing will be emphasized as well as the effects that acoustic stimuli exert on ocular activity. Furthermore, a qualitative analysis of these works will be supported by summary and comparative tables that, as previously stated, are necessary to get a synoptic vision of the features of the selected papers.

### 4.1. Demographic Characteristics of the Included Studies

In this section, an extensive review of the context and bibliography of the chosen literature is performed. More precisely, this section considers the chronology of publication activities, the geographical location of the authors who contributed to the creation of this body of scientific knowledge, the types of publication, the means of diffusion for those publications, as well as the journals and conferences most cited in this literature.

**Figure 5:**
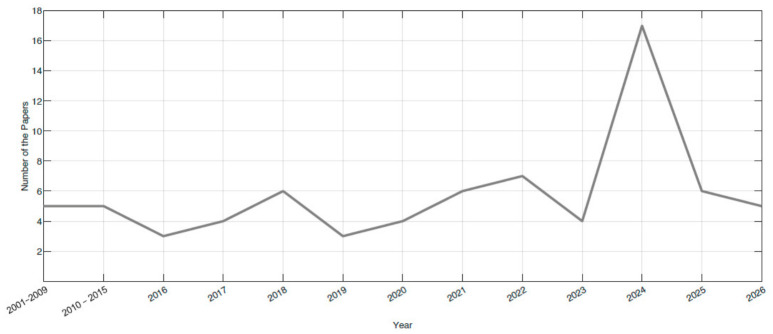
Chronological distribution of scientific publications on the integration of eye tracking and acoustics over the past twenty-five years, highlighting temporal trends, growth patterns, and key phases in the development of this interdisciplinary research field.

The first set of statistical analyses reviews the evolution of the scientific literature devoted to eye tracking and acoustics during the last twenty-five years (see Figure 5).

However, the data demonstrate that the area is evolving quite dynamically. While the early embryonic stage (2001–2015), which was featured by sparse and rather isolated research on the topic, gradually gave way to a period of rapid progress and development, in the latest three-year period (2024–2026), a true boom in scientific work was experienced. Thus, more than 30% of all papers were published during the past year, suggesting an increasing acceleration rate in scientific activities in the field.

Such dynamics suggest a change in paradigms. From the basic and fundamental research on the salience of certain sounds, science turned to the development of multi-modal sensing technologies capable of processing information from different sources. Such a change in the approach to the problem under investigation is caused by the technical advances in acoustic sensors, eye trackers, and information synthesis methods. Moreover, due to the rapid development of machine learning and deep learning, new approaches became possible.

**Figure 6:**
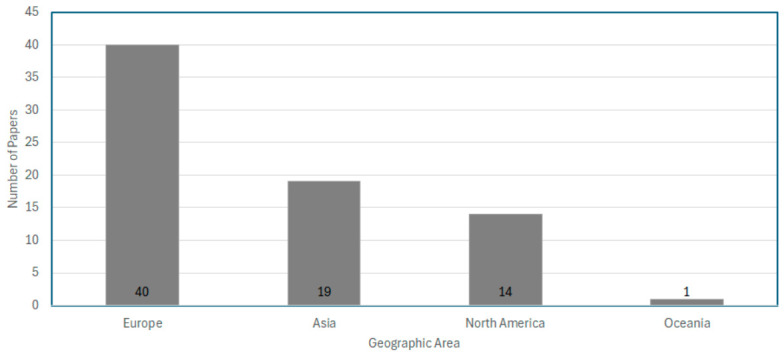
Geographical distribution of the included studies based on the institutional affiliation of the first author or predominant research team, illustrating the global contribution patterns and highlighting the region’s most actively involved in research on the integration of eye tracking and acoustics.

Finally, the second statistical analysis is concerned with the geographical distribution of studies according to the origin of their contributors (see Figure 6). The classification was performed based on the institutional affiliation of the first author or, when applicable, the predominant research team indicated in each paper. This approach provides insight into the global distribution of research activity and highlights the region’s most actively contributing to advancements in the integration of eye tracking and acoustics.

Geographical mapping of researchers’ affiliation provides an understanding of the structure of major international academic centers conducting interdisciplinary research in this domain. Europe appears to be the leading producer, contributing more than fifty percent of all scientific output; such predominance may be explained by the well-established clinical audiologists and psychoacoustics practice in the region. On the other hand, Asia plays an increasingly prominent role in the matter, particularly China with a growing number of publications directly connected with the developments in wearables and human–computer interaction. The participation of North America remains rather stable, with researchers working primarily within the frameworks of neuroscience and mobile interaction. Therefore, the current division is reflective of a globally integrated research paradigm combining medicine, engineering, and computer expertise in the field under consideration.

The second part of the analysis deals with the Typology of Publications and Dissemination Channels, which classifies articles included in the review depending on the format used for their publication (Table 1). More specifically, the classification includes established journal articles and exploratory works presented at conferences or other venues, such as books and technical reviews. This categorization helps to identify the maturity of the research contributions and the primary channels through which knowledge in the field is disseminated.

Table 1 divides the studied contributions into two types of publications: solid research papers in peer-reviewed journals and exploratory studies in scientific conferences. The overwhelming majority of publications are research papers in peer-reviewed journals (73%). This trend indicates that the methodology used in the field is becoming more stable due to a higher level of development of the field and a large amount of data obtained through experiments and clinical trials in several cases. On the other hand, a relatively large share of conference papers (24%) demonstrates the vitality of research in the field. Scientific conferences can be considered essential events in terms of communicating new technology developments quickly. These events can present experimental prototypes, datasets, and live acoustic sensing systems developed within the last period.

The last statistical analysis of the publication focuses on the top recurrent journals and conferences that play a vital role as scientific platforms and become a reference point in multimodal research that includes eye tracking and acoustics. Table 2 illustrates the main sources of scientific publications in the field.

Bibliometric references in the analysis of the most popular publication channels highlight the most important bibliometric references for research concerning the integration of eye tracking and acoustics. Analysis of data shows an extremely heterogeneous publishing environment reflecting the interdisciplinary nature of research in the field. High concentration of research papers in IEEE and ACM venues highlights the significant contribution of engineering and computer sciences to signal processing and multimodal system design. On the other hand, the regular appearance of journal papers in Hearing Research and Ear and Hearing journals indicates the clinical significance of technologies developed. Thus, there is a high likelihood that future developments in the sphere will involve collaboration between systems engineers, medical scientists, and acoustic engineers who will have to discuss their ideas and achievements in one scientific discourse.

Demographic analysis of included articles suggests that there are three major trends in the development of eye tracking and acoustics integration research. The first trend is associated with technological acceleration in the field. Almost half of analyzed research works belong to the period from 2024 to 2026. It is evident that the growing intersection between auditory sensing and eye tracking is becoming one of the key frontiers in wearable technology, powered by the progress in embedded sensing, edge computing, and machine learning-based processing of signals. Second, it is apparent that Europe dominates in terms of leading contributions to the field, owing to the established tradition in audiology, psychoacoustics, and practical studies in auditory perception. The latter aspect indicates a long history of investment into clinical and experimental studies in the field of hearing sciences.

Third, a paradigm shift towards wearables can be detected in more recent articles. While initial studies focused exclusively on laboratory settings with emphasis on sound localization, more contemporary publications tend to investigate the potential of wearable auditory technology via smart glasses or wearable interfaces for human–computer interaction.

### 4.2. Eye Tracking for Sound Source Localization and Identification

This collection of studies focuses on articles that apply eye tracking technology to determine the principles underlying the detection of sounds in space by humans. Specifically, emphasis is placed on research using eye movements to explore the processes associated with directional hearing and audio–visual integration. The importance of visual attention in constructing an auditory map is also addressed. Fixation duration, saccade movement, and eye trajectory analysis yield data on the methods used by subjects in responding to auditory stimuli presented in various directions and in the context of several simultaneous sounds. Eye tracking becomes a powerful means of examining the interaction between auditory and visual systems to enhance our understanding of the integration of multisensory cues in the brain. By reviewing past research, the key methodologies and findings are outlined in order to provide an up-to-date assessment of eye tracking capabilities in investigating sound localization and identification.

#### 4.2.1. Modeling Audiovisual Attention and Salience Mechanisms

One of the main topics explored in research on the attentional control mechanisms associated with multimodal processes is the interaction between auditory perception and visual behavior. In this context, the contributions in this paper include papers that explore the impact of auditory stimuli on gaze orientation in real and virtual contexts, paying special attention to the analysis of models of audiovisual salience. These works belong to the interdisciplinary field of cognitive neuro-sciences, psychophysics, and computer simulation. They address the problem of understanding how acoustical stimulation can affect attention allocation in a dynamical and contextual fashion. The findings reveal that acoustical stimuli do not represent an additional form of information processing, but a strong attentional bias that is able to guide the gaze outside the visual scene. More specifically, the ecological significance of certain auditory sources, such as human speech, and the precision of spatialized sounds play a crucial role in designing immersive environments and predicting visual salience.

The paper by Marighetto et al. [39] attempts to fill the lack of audiovisual salience models by proposing a new benchmark set along with a special toolkit aimed at analysis of its properties. Using eye tracking technique while observing 176 participants viewing natural video sequences, authors map the effect of sound on the exploration of faces, objects and landscapes. The offered toolkit enables calculation of various temporal and spatial indicators and allows for comparisons of eye tracking data under different acoustic conditions. In conclusion, the paper gives a useful methodology for evaluating computational models of attention with proper audio integration into the process.

The article by Song et al. [40] discusses the role played by different categories of sounds in eye-gaze regulation while viewing freely-chosen audiovisual sequences. Through comparison of purely visual and audiovisual condition the researchers conclude that human voices (speech and singing) have the biggest impact on the gaze behavior of the observer. Additionally, the paper shows that not all sounds can be classified as attractors and draw the eyes towards their source, and that the presence of sounds generally leads to higher rate of fixation transitions and lower time on fixations.

The study by Xue et al. [41] seeks to solve the problem of narrative management in Cinematic VR (CVR), in which the ability to move freely might break the flow of the narration. The study assesses the efficiency of using spatial audio as an attention control (CUE) toward events that take place beyond the visual field. Using the eye tracking technique in less complex virtual reality environments, it is revealed that viewers have a better reaction to audio CUEs that differ horizontally rather than vertically.

**Figure 7:**
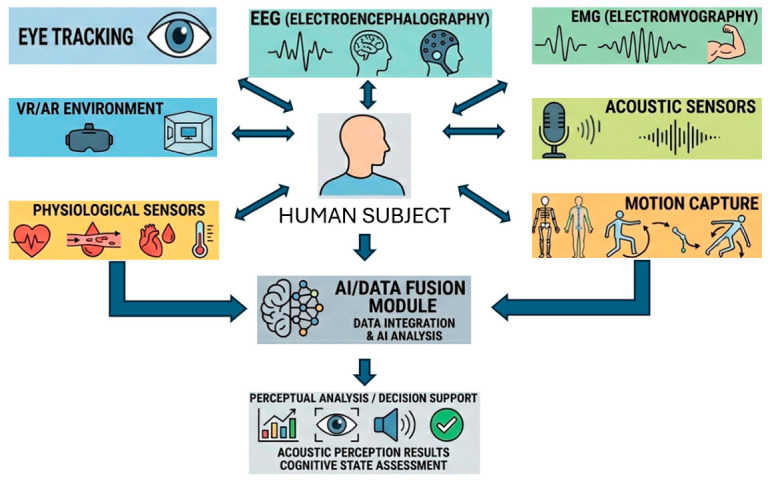
Multimodal AI-based data fusion architecture for human-subject monitoring and perceptual assessment. The framework is organized into three interconnected layers: Data Acquisition Layer comprises the primary sensing modalities surrounding the participant, including neurological measurements (EEG), behavioral monitoring systems (eye tracking and motion capture), physiological sensors (e.g., heart rate, skin conductance, and EMG), immersive VR/AR environments, and acoustic sensing technologies. These complementary data streams capture different aspects of the participant’s cognitive, physiological, behavioral, and environmental interactions. Data Integration Layer contains the AI/Data Fusion Module, which synchronizes heterogeneous data sources, aligns multimodal features in time and space, and applies artificial intelligence algorithms to extract meaningful patterns and relationships across sensing modalities. Analytical Output Layer represents the interpretation and decision-support stage. At this level, the fused information is transformed into actionable outcomes, including acoustic perception metrics, behavioral indicators, and comprehensive assessments of cognitive state, enabling a deeper understanding of human responses to complex acoustic and environmental stimuli.

To synthesize the various behavioral, physiological, and contextual data streams discussed previously, Figure 7 outlines the comprehensive multimodal data fusion architecture proposed for holistic human-subject monitoring. This framework serves as the operational backbone for integrating heterogeneous sensor data, transitioning from raw physical and biological metrics to actionable cognitive and perceptual insights.

At the core of the architecture is the Human Subject, who simultaneously interacts with the environment and exhibits measurable physiological responses. The input layer consists of seven distinct monitoring and contextual modalities, categorized by their data collection objectives:

- Neurobehavioral and Spatial Inputs: Eye Tracking captures gaze dynamics and fixations; EEG (Electroencephalography) tracks cortical activity and neural oscillations; and the VR/AR Environment provides context regarding the digital or augmented spatial surroundings.
- Physiological and Biomarker Inputs: Physiological Sensors monitor autonomic responses (e.g., heart rate, blood flow, and skin temperature), while EMG (Electromyography) records muscular tension and micro-movements, reflecting physical manifestations of stress or orientation effort.
- Environmental and Kinematic Inputs: Acoustic Sensors track the ambient sound fields and audio stimuli, while Motion Capture systems log whole-body kinematics and postural adjustments.

The true utility of this architecture lies in its processing and abstraction layer. All synchronous data streams are routed into a centralized AI/Data Fusion Module. This module handles the complex challenges of cross-modal synchronization, feature extraction, and machine learning-driven pattern recognition. By combining low-level physiological signals with high-level environmental context, the system progresses to the final output phase: Perceptual Analysis/Decision Support. This layer delivers quantified assessments of Acoustic Perception Results and an ongoing Cognitive State Assessment, allowing researchers or clinical systems to objectively evaluate listening effort, stress levels, and spatial awareness in real time.

Roßkopf et al. [42] investigated the effectiveness of binaural auralizations within a virtual university classroom, comparing different rendering techniques (based on simulations or real measurements) with listening through physical speakers. Through spatial positioning and gaze tracking tasks, the study reveals that, although binaural rendering does not yet achieve the millimeter-level precision of real speakers in localization, it does ensure equivalent levels of social presence and subjective realism. Significantly, personalized simulations significantly improve the feeling of social participation compared to generic ones. In summary, the implementation of plausible binaural audio models is considered essential to optimize immersion in collaborative or therapeutic VR contexts.

In a further study, Roßkopf et al. [43] analyzed the effectiveness of binaural auralizations with head tracking in improving social presence and localization accuracy in Virtual Reality (VR) contexts. Using eye tracking as a tool to measure spatial attention, the researchers compared realistic audio renderings with physical speakers and standard video game audio engines. The results show that high-quality auralizations achieve sound externalization rates close to 100%, with distance perception comparable to that of real sources. However, as seen by the high correlation between audio quality and the level of social presence, precise acoustic reproduction is essential in applications that require it. In particular, exposure therapy can benefit greatly from authentic immersive experiences provided through correct reproduction of the acoustics in order to elicit natural reactions from users.

Król [44] examines the effects of environmental noise on the visual strategies employed by children in exploring faces in terms of the attentional trade-off that arises between social and linguistic aspects. As predicted, the data obtained show that acoustic distraction causes participants to look at the mouth rather than the eyes because of difficulties in processing linguistic cues visually. However, in doing so, they also decrease the time spent tracking the eyes and, thus, lose valuable socio-emotional information. Moreover, the research finds that higher proficiency in language and high levels of pupil dilation (a marker of attention and motivation) correlate positively with looking at the mouth.

Galanda et al. [45] introduce a novel approach to sound source localization, using eye movements for this purpose and implementing it with the help of a standard webcam. Based on the premise that the gaze naturally tends to align itself with acoustic cues, an eye tracking technique has been formulated which incorporates facial landmark tracking as well as Kalman filtering to account for the effects of head motion and filtering of signals. Validation, based on reaction time studies, has shown that the technique can be applied in high-risk scenarios like aviation safety, airport noise monitoring, and car driving control.

The study by Gehmacher et al. [46] reveals a common neural pathway between attention and eye movements, leading to the concept of “speech eye tracking”. Using eye tracking in conjunction with magnetoencephalography, it has been observed that the gaze naturally follows the speaker being attended to. These data demonstrate that oculomotor activity actively contributes to neural responses during listening. The study therefore encourages us to consider eye movements as a central, rather than marginal, element for understanding cognitive processes related to hearing in ecological contexts.

Table 3 below summarizes the studies analyzed, listing for each contribution the authors, sample considered, technologies used, and main findings related to audiovisual salience.

The findings described in Table 3 demonstrate the importance of auditory information in determining audiovisual attention and salience processes. Many papers stress the necessity of developing specific databases and toolkits aimed at investigating the effect of sound on visual salience. In general, auditory stimuli greatly facilitate visual search, leading to an increase in eye movements. Specifically, human speech turns out to be one of the most salient stimuli and, hence, attracts visual attention. Spatial sound is also helpful in guiding the gaze direction and even moving eyes outside the direct line of sight, particularly in the horizontal direction. The application of binaural audio and realistic acoustic models, such as HRTF-based approaches, boosts the sense of realism and social presence and makes it possible to localize sounds accurately.

#### 4.2.2. Applications in Clinical and Diagnostic Contexts

**Figure 8:**
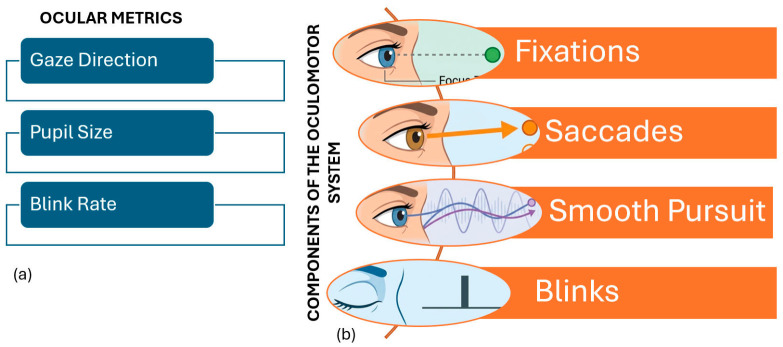
Conceptual mapping of core ocular metrics to physiological oculomotor system components. (**a**) Ocular Metrics: Outlines the three primary data streams captured by eye-tracking hardware: Gaze Direction, Pupil Size, and Blink Rate. (**b**) Components of the Oculomotor System: Visualizes the corresponding physiological behaviors and kinematic signatures. These include Fixations (static focus maintenance), Saccades (ballistic spatial transitions), Smooth Pursuit (continuous trajectory tracking), and Blinks (eyelid occlusion intervals), which serve as key behavioral indicators of cognitive load, visual attention, and spatial orientation performance.

Eye tracking and sound stimulation have proven to be a promising technology when used for the detection and analysis of various health conditions. More specifically, in clinical settings, eye tracking and sounds can assist in detecting developmental disorders at an early age and monitor the psychophysical state of people in real-time. The literature examined within the current paper uses a multimodal approach to combine visual, audio, and physiological information to provide a better understanding of human emotions and cognitive processes. As a result, these models allow overcoming the limitations of single data input by providing much greater predictive performance. On the one hand, it was demonstrated that synchronization of auditory and visual information significantly impacts linguistic development; at the same time, there were some differences depending on the gender of the person under study. On the other hand, physiological information provided through eye tracking (for example, pupil diameter) could be successfully applied to the detection of stressful conditions better than any other measure. To ground the subsequent analysis in physiological fundamentals, Figure 8 delineates the primary oculomotor mechanics activated during auditory processing. It tracks how foundational eye movements—namely stationary fixations, rapid saccadic jumps, and continuous smooth pursuit tracking—are dynamically modulated by acoustic properties and shifting psychological states. By synchronizing these gaze metrics along a unified timeline alongside varying stimulus intensities, discrete audio events, and evolving cognitive workloads, the diagram highlights the intricate sensorimotor coupling that occurs during auditory tasks (Figure 8a). Ultimately, it serves as a conceptual bridge, demonstrating how raw ocular behavior translates into measurable indicators of directional hearing and focused attention.

The system in Figure 8b maps three primary categories of measurable data to four specialized oculomotor behaviors:

- Gaze Direction mapping to Fixations and Saccades: Gaze Direction provides the foundational spatial coordinates of visual attention. When a subject identifies a target, the oculomotor system stabilizes the eye via a Fixation, maintaining a steady focus on a singular point to process information. Conversely, when shifting attention between targets—such as moving toward an acoustic source—the system executes Saccades, which are rapid, ballistic eye movements designed to ballistically re-orient the fovea.
- Gaze Direction mapping to Smooth Pursuit: When a target or sound source is moving continuously through space, the oculomotor system transitions to Smooth Pursuit. Unlike saccades, smooth pursuit allows the eyes to track an object seamlessly without interruption, serving as a vital metric for studying dynamic audio–visual tracking performance.
- Pupil Size and Blink Rate mapping to Blinks: Independent of directional movement, Pupil Size and Blink Rate provide direct insight into the user’s internal mental state. Fluctuations in pupil diameter serve as a well-documented proxy for autonomic nervous system arousal and listening effort, while Blinks and overall blink frequency are heavily correlated with cognitive fatigue, processing load, and situational stress.

By isolating these individual oculomotor components, researchers can dissect not only where a subject is looking, but how much mental energy they are expending to analyze their environment.

The study by Zhang et al. [47] offers a supervised learning model to aid early detection of autism spectrum disorders (ASD). Focusing on analysis of visual scan paths and voice cepstral coefficients (MFCCs), the researchers developed advanced predictive models (XGBoost and neural networks) based on a study cohort of 108 children. The multimodal method proved its ability to diagnose ASD with an 82% rate accuracy, demonstrating better results than methods using only one modality. In other words, the use of multiple bio-metric signals appears to be an efficient, scalable, and affordable way of developing predictive models.

The study by Clark et al. [48] examines the influence of visual attraction to audio–visual synchronous speech on linguistic development in children aged 12–18 months old, contrasting the behavior of those exposed to high risks of autism with control subjects. Eye tracking experiments demonstrated that, unlike their counterparts, control participants had a higher attraction to synchronous audio–visual speech stimuli. However, despite no direct connection between preferences and expressive language, further analysis showed that the preference increased complexity of vocalization among male children, thus enhancing linguistic capabilities. In this regard, the study emphasizes the role of multisensory perception in developing language skills.

Speech recognition is a task affected by several variables, including environmental noise, foreign accents, or hearing loss, which makes this process mentally difficult despite understanding spoken language. The work of Van Engen et al. [49] suggests that eye tracking and pupillometry be used to receive real-time objective data about effort. While eye tracking helps to understand visual focus during listening, pupillometry gives physiological measure of mental load in terms of changes in pupil size. Thus, these methods give researchers a chance to understand adaptation mechanisms applied by listeners in everyday communication tasks.

The novel method for mapping sound localization using eye tracking introduced by Volck et al. [7] provides a fast and easier way of measuring hearing compared to other instruments available today. Testing of this instrument involved fifteen participants, and the results showed very high repeatability and reliable correlations between visual reactions and auditory stimuli. The sensitivity of the instrument was tested on hearing impairment simulation, producing systematic error of around 5.5° towards the healthier ear. Overall, the approach can be considered as a viable and reliable choice to assess the spatial hearing abilities, providing outcomes equivalent to costlier methods but within shorter timeframes.

Table 4 provides a brief overview of major studies examining clinical and diagnostic uses of eye tracking technology. The table shows the importance of eye movements to evaluate auditory processes, cognitive loading, and multisensory integration. As can be seen from the outcomes presented, eye tracking technology has significant potential to serve as a non-invasive tool in clinical diagnostics.

The outcomes shown in Table 4 reveal the efficiency of multimodal approaches for improving not only diagnostic precision but also interpretative proficiency. Specifically, the use of several types of data leads to 82% precision, which is higher than those from mono-modal approaches. The combination of auditory and visual information is particularly vital for the diagnosis process. In addition, according to behavioral evidence, a predisposition to synchronous speaking acts as a mediator of vocal complexity, especially for male patients, which might be considered for linguistic research. Lastly, physiological indicators like pupillary diameter are effective predictors of stress with 79.2% precision, surpassing electrodermal activity measurements.

#### 4.2.3. Speech Processing and Hearing Assistive Technologies Eye, Acoustic, and Neural Signals Integration: Future Directions for Speech Processing and Intelligent Hearing Devices

This section includes research papers that investigate the potential benefits of incorporating eye tracking technologies into the process of speech selection and understanding. In particular, the studies described below aim at demonstrating that eye movements may become an integral part of the process of separating and reconstructing speech signals in complicated conditions, when several sound sources are involved. According to the authors, gaze is not just an additional parameter for assessing attention; on the contrary, the information derived from the analysis of eye movements can become one of the components of the signal selection process. For instance, one can train machine learning models on the basis of labeled data, which can be easily obtained using eye trackers, resulting in the increase in signal-to-noise ratio. Neural activity data reveal that the process of speech tracking is tightly related to the trade-off between intelligibility and attention costs. Finally, the connection between eye movements and speech rhythm demonstrates that visual and auditory systems share some neuro-mechanisms.

The investigation carried out by Wilroth et al. [50] addresses the issue of the combination of eye tracking and auditory attention decoding (AAD) with the application of mobile EEG technology, seeking to develop future-proof hearing aid designs. Specifically, it was shown that it is possible to rely on eye movements to automatically determine the sound source to which the subject is paying attention, thus producing effective labels needed for speech reconstruction training. While the tests were quite preliminary in nature, the results obtained confirmed that the development of a non-manually supervised brain-operated system is entirely feasible, offering significant benefits for the filtrations capacities of hearing devices used under noisy conditions.

Finally, the research by Grimm et al. [51] presents a more elaborate solution for speech enhancement in the hearing aids, combining spatial filtering with the determination of gaze orientation. With the use of both electrooculography and head tracking techniques, the system detects the sound source to which the subject is paying attention, thus identifying the position of each interlocutor in the complex acoustical environment. Tests performed with the participation of fourteen subjects have demonstrated that gaze-based automatic audio filtering provided an SNR improvement of up to 7 dB.

The study by He et al. [52] focuses on mechanisms of selection and tracking of speech by the brain in a noisy environment, and shows that there is a non-linear correlation between SNR and neural activation. With the help of EEG and eye tracking, it was revealed that neural tracking of target speech declines with an increase in signal enhancement; conversely, when listening becomes easy, it gets worse. The study uses gaze velocity as the measure of attentional effort (AE) and behavioral tests for measuring signal intelligibility (SI); hence, it can be concluded that neural tracking depends on the two opposite factors: although enhancement of the signal favors it, the decreased attention needed to decode it weakens it. Thus, this work shows that SNR has no direct effect on brain activity; however, it acts as a mediator of the balance between the signal quality and listener’s cognitive load.

The study by Eloy et al. [9] investigates how vision and hearing work together in the exploration of an environment in both physical reality and virtual environment. Conducted in the context of a university campus using eye tracking method, the research shows that audio stimulus is essential to achieve behavioral fidelity in terms of: virtual replicas that include sound generate responses much more similar to those in the real world than purely visual ones. Acoustic input is not just a contour, but a determining factor in spatial perception, essential for validating virtual reality as a behavioral research tool.

Oculomotor dynamics while listening and perceiving verbal communication have been examined in studies by Lansing et al. [53], where the focus lies on two factors—ocular primacy, which stands for preference for fixing one’s eyes when at rest, and attraction to the source of the incoming information, meaning eye movement towards the mouth area. It was discovered that with the onset of speech there occurred saccade eye movements directed to the mouth area accompanied by simultaneous eye suppression in order to facilitate processing. In spite of the tendency to focus on the mouth becoming higher with task difficulty, the final decoding accuracy depended on individual proficiency in visual decoding rather than the time spent on gazing at particular facial features.

In their paper, Vasilev et al. [54] investigate how cognitive conflict arises in reading comprehension due to auditory interference, making a distinction between the processes of lexical access and semantic integration. Results obtained through the course of three experiments showed that intelligible speech does not obstruct instantaneous word recognition, yet greatly increases regressions and re-reading rate. This observation suggests that distraction is semantic and post-lexical rather than phonological. Crucially, comprehension declines only if rereading is prevented, demonstrating how eye movements serve as an essential compensatory strategy to mitigate the semantic interference of verbal noise.

The work of Kanerva et al. [55] focuses on the semantic decoding capacity of Russian onomatopoeia in Finnish participants who lack particular linguistic skills to test the hypothesis of the universality of sound symbolism. Using an initial free association task, authors managed to categorize responses into clusters—“facilitating,” “contrasting,” “mixed,” and “undefined” clusters—that emphasize the importance of particular acoustic features in guiding the perceiver in the right direction or misleading them intentionally. In the latter experiment performed by eye tracking method, the presence of iconic cues significantly increased recognition accuracy. Summing up, findings demonstrate that onomatopoeias serve as an “interlinguistic bridge” due to common form-meaning associations, providing evidence of the universal imitative nature of language.

Table 5 summarizes studies investigating eye tracking in speech processing and hearing assistive technologies. It highlights how gaze behavior reflects speech intelligibility, listening effort, and device performance. The findings emphasize the role of eye tracking as an objective tool to evaluate and optimize hearing aids and related auditory support systems.

Key findings presented in Table 5 emphasize the close relation between gaze behavior and speech processing, especially when hearing assistance technology is used. Eye tracking can be an effective method for automatic labeling of audio signal sources, helping with a better efficiency of learning how to reconstruct speech. Additionally, gaze-guided filtering improves the quality of hearing significantly, raising the signal-to-noise ratio (SNR) even by 7 dB. It has been demonstrated that neural tracking of speech is affected by the compromise between the understandability of speech and attention costs. Finally, the coordination of gaze and speech rhythm implies the presence of common neural mechanisms behind the two phenomena.

#### 4.2.4. Attention Patterns and Professional Skill Assessment

The combination of various types of attentional states and the evaluation of soft skills constitutes an increasingly popular topic in the intersection of eye tracking and acoustic analysis. This chapter includes studies that aim at detecting the presence of different types of attention, namely sustained attention, selective attention, alternating attention, and divided attention, along with the potential for evaluating cognitive and relational skills based on behavioral and physiological markers. It is evident from these studies that the multimodal approach results in high classification accuracy, revealing that attention states can indeed be reliably classified based on eye movements in conjunction with acoustic and physiologic markers. Specifically, gaze direction is found to be the main parameter for measuring empathy, which implies the necessity to maintain constant visual contact in communicative settings. On the other hand, there are cases where the effectivity of using heart rate is proven superior to that of the eye movements, particularly in cases where one is listening to audiovisual content.

Abdelrahman et al. [56] proposed a groundbreaking methodology for non-invasive discrimination among four forms of attention: sustained, alternating, selective, and divided attention. Using a combination of thermal imaging and eye tracking, the researchers examined responses from twenty-two participants subjected to audiovisual stimulation. Applying logistic regression models, the algorithm succeeded in classifying the four forms of attention with a very high degree of accuracy. It is demonstrated how the interplay of facial thermoregulation and eye movements may reflect cognitive processes and pave the way for future developments of interfaces reacting to the attentional state of the user in real time.

Ito et al. [57] propose a protocol to quantify the empathy of nurses using eye tracking, speech recognition and recording videos. The researchers used a simulation of active listening to map eye movements and found out that nurses were looking at their patients’ faces 94% of the time, with special emphasis on the right eye region and mandible. In addition, acoustic analysis enabled them to find associations between pressure changes and emotions like laughter and utterances of agreement. In short, the protocol transforms empathy—often considered an intangible quality—into numerical and visual data, providing a rigorous tool for training and assessing relational skills in the clinical setting.

According to Hartnett et al. [58], the role of eye tracking in measuring attention to advertisements as a unique variable was challenged, and the researchers suggested applying several physiologic variables. In an experiment that varied the level of attention to the videos (high/low), it became clear that eye tracking failed to discriminate between attention intensity variations. Meanwhile, heart rate turned out to be the best metric that could capture attention toward both visual and auditory cues of the ad. Summarizing, this research shows that assessing the quality of exposure to advertisements in a reliable manner would require using more physiologic parameters reflecting the level of effort.

Lehtilä et al. [59] have explored utterance fluency of bilingual and multilingual speakers (L1, L2, L3) applying eye tracking for mapping the cognitive processes underlying. Analyzing Finnis speakers during picture descriptions, the researchers have revealed that the level of fluency is not uniform for everyone, especially when it comes to managing hesitations. Eye tracking data offer a valuable window into language planning, showing how gaze reflects mental effort during oral production. These findings suggest that integrating eye tracking could revolutionize language assessment and teaching, allowing for a deeper understanding of multilingual communicative competence.

Doyle et al. [8] demonstrated that the synchronized presentation of uninformative acoustic cues accelerates the identification of visual targets without sacrificing accuracy. This facilitation appears extremely robust, as it does not depend on spatial coherence between sound and image, stimulus contrast, or the subject’s eye movement. The authors suggest that auditory input promotes faster attentional disengagement, optimizing neural processing times. In summary, the mere temporal co-occurrence of an accessory sound acts as a cognitive enhancer, overcoming the limitations of the visual modality alone in monitoring the surrounding environment.

Gerdes et al. [60] analyzed the cross-modal interaction between auditory stimuli and emotion in the management of visual attention. Using a free-viewing paradigm paired with auditory stimuli, the researchers demonstrated that the valence and position of sounds influence eye orienting toward unpleasant images. Specifically, spatial congruence between sound and image enhances both initial and sustained attention. Furthermore, negatively charged sounds accelerate pointing toward emotionally coherent images, suggesting a multisensory attentional prioritization mechanism. Emotional auditory cues act as powerful spatial catalysts, effectively directing the gaze toward visual content with the same affective relevance.

The research by Yang et al. [61] introduces a museum experience system that integrates paintings and spatialized audio, personalizing the audio experience through gaze tracking. Associating specific sounds with depicted elements allows visitors to deeply immerse themselves in the scenes, while eye tracking ensures that the audio dynamically responds to the subject’s visual behavior. A study conducted on fourteen participants confirms that this multisensory interaction enhances concentration on areas of interest, significantly improving the overall aesthetic experience. In short, the technology transforms the static viewing of the painting into an active multisensory exploration, capable of guiding attention in an intuitive and engaging way.

The research conducted by Baumann et al. [62] examines the neural basis for SPEM in conjunction with the tracking of auditory stimuli. This experiment aimed at investigating the way in which the brain processes cognitive load in a situation where gaze and auditory attention are directed differently. An audio stimulus was used as a distractor, allowing separating the attentional conflict from any kind of visual distraction. According to the findings, there is a higher activity of the parietal and frontal lobes where eye fields reside, indicating that the process of coordinating conflicting sensory flows entails huge engagement of attentional mechanisms.

Table 6 below is a summary of various studies examining attention patterns associated with professional skills assessment through the use of eye tracking methods. It highlights how visual behavior reflects expertise, cognitive strategies, and task efficiency across different domains. The reported findings show that gaze metrics can effectively discriminate skill levels and support objective evaluation of professional performance.

The results summarized in Table 6 highlight the potential of multimodal approaches for classifying attention states and assessing professional skills. In particular, eye tracking-based models are able to distinguish between sustained, alternating, selective, and divided attention with high performance, reaching AUC values up to 87%. Additional findings show that gaze behavior can be effectively used to infer socio-cognitive attributes such as empathy, with participants allocating up to 94% of visual attention to the face, modulated by variations in sound pressure. However, physiological measures such as heart rate can outperform eye tracking in certain contexts, such as differentiating levels of attention to video advertisements. Finally, gaze dynamics provide insights into cognitive effort and production difficulties, revealing stalling mechanisms during language production tasks across different linguistic contexts.

Overall, the reviewed studies consistently highlight that eye-tracking metrics—particularly fixation behavior, saccade patterns, and gaze trajectories—provide converging evidence that visual attention plays a critical role in auditory spatial processing, although methodological differences across experiments limit direct comparability of results and point to the need for more standardized experimental paradigms in future research.

### 4.3. Eye Tracking in Sound Event Detection and Classification

In recent years, the integration of eye tracking technologies and advanced computational models has opened new perspectives in the field of Sound Event Detection and Classification (SEDC). In this context, eye movements are no longer considered exclusively as behavioral indicators, but become a complementary source of information to improve the performance of automatic sound analysis systems. In particular, visual attention can provide relevant clues about the perceptual salience of sound events, helping to guide machine learning and deep learning algorithms in the training and inference phase. Several studies have demonstrated how integrating eye tracking data with audio and, sometimes, audiovisual signals allow for the development of more robust and interpretable multimodal models. Such approaches exploit, for example, fixation maps or eye trajectories to dynamically weight the importance of specific components of the acoustic signal or to improve the localization and classification of sound events in complex environments. Moreover, eye tracking can help in developing annotated data sets in a much better way by avoiding the uncertainties in manually annotated data sets. This part of the study will discuss the significant contributions made in literature with respect to eye tracking and computational models used for detecting and classifying sound events.

#### 4.3.1. Computational and Technological Frameworks

In this part, the technological advancements in the field of eye tracking will be discussed, paying particular attention to the most promising technologies that increase the scope of applications of eye tracking in multimodal contexts. First of all, the advancement in sensor technologies will be reviewed, especially in terms of new event-based sensors, which represent an important development in relation to frame-based sensors owing to their capability to detect changes in visual input at a high sampling rate with low power consumption. Event-based sensors are therefore perfectly suited for real-time applications that require quick reaction times. Moreover, there is an increasing demand for the standardization of eye tracking processes in order to ensure interoperability across different platforms. The creation of common data formats for acquisition and processing of eye tracking signals ensures replicability of experiments as well as the possibility of data integration with other kinds of data like acoustic inputs and contextual information. Finally, the use of artificial intelligence algorithms, particularly machine learning, constitutes a key technology in saliency prediction in multimodal environments.

Nurlatifa et al. [10]’s paper explores the importance of the eye as the primary interface between humans and computers, contributing significantly to the improvement of eye tracking technology. The main idea discussed by the authors is what is called event detection, an operation involving eye movements that plays an important role in improving the standards of tracking systems. In particular, the paper considers various technical features mentioned frequently in the scientific literature on eye movement analysis: sampling rate, type of stimuli, video tracker design, and signal noise reduction techniques. Furthermore, besides describing the development of research trends over re-cent years, the authors discuss methodological problems faced by scholars at present. Finally, the goal of their paper is to offer a systematic review useful for scientists studying the topic of eye event detection.

Event-based eye tracking constitutes a significant breakthrough that will make modern smart glasses more energy efficient. Unfortunately, the deployment of event-based sensors is hindered by the lack of annotated data sets. With regard to this aspect, the research carried out by Simpsi et al. [11] brings a significant update to the benchmarking datasets of the field. The major contribution brought by the research is embodied in the creation of the semi-automatic pipeline for the annotation process, which is particularly designed to deal with the data generated by the use of event-based sensors. By doing this, the researchers make possible the provision of new and accurate annotations for the detection of pupils, making available an excellent scientific tool to improve eye tracking algorithms.

Concerning the topic of predicting attention salience for multimedia environments, it is important to note that in most cases, the role played by the interaction between auditory and visual stimuli is not taken into consideration. In order to solve this problem, the study conducted by Qiao et al. [63] introduces AVM-Net, a multitask model capable of performing tasks of audiovisual salience detection and sound localization of videos with multiple faces. Backed up by an innovative database that links eye tracking and audio annotations, the research illustrates the reciprocity existing between sounds and gazes: while sounds drive gazes, the opposite is also true. Each branch is dedicated to processing spatial, temporal, acoustic, and physiognomic information individually, while multimodal spatiotemporal graph combines them together. The simultaneous optimization of these tasks enables the model to surpass twelve current approaches, thus proving that the integrated approach helps understand the dynamics of attention in complex environments effectively.

Despite multimodality of video media, most of the models for visual attention modeling developed in the recent decade tend to ignore the effects that sound might have on people’s gaze behavior. Sidaty et al. [64] presented their audiovisual saliency model which was aimed to predict human fixations on video calls using spatial, temporal, and auditory attention maps. The novelty of this study is in its real-time speaker localization system that adjusts the auditory map depending on whether the particular face is the active speaker or a listener. The superiority of the approach can be easily traced when comparing advanced performance measures of the model with eye tracking data. The research thus confirms that multimodal fusion is essential for an accurate representation of human attention in digital communication contexts.

Table 7 summarizes the main methodological contributions in the literature, highlighting the most recent hardware and software innovations for the integrated processing of ocular and acoustic data. Particular attention is paid to multimodal acquisition, synchronization, and analysis techniques, as well as their application implications.

This can be seen in Table 7, where there is an evident progression in the techno-logical and methodological landscape of this study. The most evident feature is that it shows how the methodology and approach to attention research changed from the traditional methods, mainly based on vision model-based techniques, towards more integrated solutions, such as those presenting high-energy efficiency and growing computational complexity. Particularly noteworthy is the use of advanced neural architectures in order to better simulate the attention process. In this regard, a very important aspect that arises concerns the use of specific databases that contain synchronized multimodal data. In fact, it is thanks to the availability of this type of database that more sophisticated and robust models could be generated. In fact, this approach allowed overcoming the limits of monomodal models that, by their very nature, fail to take into account all the complexity of human attention. This becomes particularly important when it comes to adding the audio input. Not only does it help provide new information but also helps understand perception in digital and communicative environment better.

#### 4.3.2. Neurophysiological Mechanisms and Cognitive Interaction

This section provides an in-depth analysis of the relationship between auditory perception and oculomotor function, attempting to highlight the influence of different neurological and physiological variables on the process of sound localization. The interaction between the auditory system and the visual system represents an essential part of orientation and attention mechanisms, where eye movements represent not just a consequence of the direction of gaze but also become an active component of the scene construction. In this context, particular emphasis should be placed on cognitive load, which can affect the accuracy and the speed of eye movements, indirectly affecting sound localization tasks. In addition, different levels of consciousness can impact multisensory processing mechanisms by influencing the synchronization between sensory inputs and motor actions. Finally, vestibular reflexes represent another relevant aspect, since they guarantee gaze stabilization when moving, thereby ensuring coordination between auditory perception and spatial reference.

Based on the analysis of recent works, this section illustrates how these aspects help define the accuracy and reliability of sound localization mechanisms.

In the paper by Pomper et al., [12], the intricate connection between gaze orientation and auditory attention is explored from both behavioral and physiological perspectives. Through use of EEG, the authors studied instances where visual orientation was manipulated to be incongruent with the sound source. The findings show a clear impairment in speed of responses whenever gaze direction did not match the sound source. Neurologically, this mismatch was linked with an increased power in alpha waves in the contra-lateral occipital region, seen as a strategy to reduce interference from irrelevant visual input. At the same time, there is a marked increase in activity in the theta band from the central region, pointing to greater effort in top-down attentional control. In essence, research suggests that disengaging gaze direction from auditory attention reduces processing efficiency, but triggers specific neuronal processes to counteract the adverse effect of non-relevant cues.

The study by Wang et al. [65] examines the ability of the brain to localize sound—a higher level cognitive function usually performed in the posterior auditory cortex—during NREM sleep. In their study, Wang et al. used the frequency tagging method using simultaneous recording of EEG and magnetoencephalography (MEG) techniques. The findings showed a marked difference in the processing abilities of the brain. Although neural activity involved in lower-level processing of sound is still observable even during sleep (though lessened), the brain no longer produces specific neural responses to sound location during NREM sleep. It implies that, despite the capability of the sleeping brain to perform basic sound analysis, other higher brain functions needed to localize the sound source are no longer present.

In turn, the research conducted by Sarac et al. [66] examines the interrelation of the vestibulo-ocular reflexes and the auditory cognitive load, studying the effect of “what” (recognition) and “where” (location) tasks on the control of eye movements. With the help of videonystagmography (VNG), the authors have studied the performance of saccades and slow pursuit when no exogenous stimuli were present, during the identification of sounds, and their location. It is clear from the data obtained in the experiment that the execution of any cognitive task adversely affects the results in all ocular motor parameters recorded by means of VNG. Specifically, it is revealed that the necessity of processing auditory stimulation is a distracting element that reduces the accuracy of visual scanning. Such an effect shows the influence of the “dual task” performance characteristic for our everyday life.

The audiogyral illusion is another auditory illusion where a stationary sound source in relation to the head is perceived to move as the body rotates in darkness. It causes an erroneous movement of the subjective auditory median plane (AMP). Van Barneveld et al. [67] studied whether such an AMP displacement could be related to vestibular cues, fixation of the eyes or eye movements by rotating subjects either in darkness or with a fixation light. Results show that darkness rotation leads to an AMP displacement, whereas AMP displacements disappear when there is a visual fixation light. Moreover, eye-pursuit experiments revealed that AMP displacement occurs when there is eye eccentricity but not when there is eye motion velocity. From this study, it is clear that AMP shift and audiogyral illusion are neither caused by vestibular rotation nor by vestibular-induced eye movement velocities but rather by mean eccentricities of the eyes created by vestibular inputs.

Contemporary geographical information systems (GIS) rely on multifaceted data flows requiring significant cognitive work, hence impairing the timely detection of spatial signals under critical circumstances. Zhang et al. [68] evaluated the role of spatial acoustic signaling in enhancing the localization of events in crowded surveillance displays. An empirical study conducted with 24 subjects showed that display dynamics, interface complexity (12 panels or more), and varied forms of auditory stimuli (binaural vs. monaural) have a substantial effect on the process. Based on eye tracking measurements and performance scores, researchers found that high visual complexity adversely affects performance, whereas lateralized acoustic signals enhance event localization. More specifically, binaural cues offset the cognitive burden associated with dynamic maps, whereas monaural cues compensate for visual clutter in complex interface designs. This study shows how integrating spatial audio cues promotes situational awareness in GIS systems, providing guidance for the development of ergonomic multimodal interfaces that do not solely rely on visual stimulation.

Table 8 provides an overview of the principal correlations identified in brain activity (EEG/MEG), vestibular functions, and ocular behavior in response to acoustic stimuli.

The analysis reported in Table 8 clearly highlights how the oculomotor system plays an active and integrated role in perceptual processes, overcoming the traditional view that considers it a simple motor executor. The data show that eye movements sensitively reflect the subject’s cognitive effort and neural state, emerging as reliable indicators of the internal dynamics of sensory information processing. Particularly significant are phenomena related to multisensory integration, such as the audiogyral illusion, which demonstrates how the perception of sound motion can be influenced by the coordination between auditory inputs and oculomotor signals. Similarly, the suppression of sound localization during sleep highlights a reduction in coherence between the sensory and motor systems, suggesting that the construction of the auditory map is strongly dependent on the state of brain activation. These results highlight a profound interdependence between ocular proprioception and auditory perception, indicating that the spatial representation of sound is the result of a distributed and dynamic process. Overall, the table highlights the importance of considering integrated approaches to fully understand the mechanisms underlying sound localization.

#### 4.3.3. Clinical, Developmental, and Prosthetic Applications

This part of the paper aims at examining the principal uses of eye tracking technology in the field of audition, especially the study of the emergence of perceptual skills in pediatric populations and the evaluation of the efficacy of different types of hearing prosthetics. Eye movement assessment is a non-invasive yet very revealing technique that is used in audiological studies in order to examine the onset of auditory processing, which is particularly useful when no verbal responses are available. Simultaneously, eye tracking is a novel diagnostic method that may be employed in the assessment of the efficiency of artificial hearing aids, such as cochlear implants and bone conduction hearing devices. By assessing the eye movement characteristics of patients, the clinician receives valuable insights into the capacity of the individual to interpret auditory stimuli, thus making it easier to optimize prosthetics in accordance with each person’s needs. The combination of ocular movements and acoustic information opens up new possibilities for diagnosing hearing impairments.

Alemu et al. [69] have researched the synchronization between the motion of the head and eyes during sound localization and compared the behavior of children with adults. The experiment explored how static and dynamic acoustic objects were localized while limiting binaural cues by blocking one ear temporarily. Based on the analysis conducted through eye tracking glasses and gyroscopic sensors, the researchers concluded that, in case of normal auditory capabilities, ocular motion preceded head motion. However, when aural occlusion was introduced into the test, it seemed that the subjects were compensating for the lack of hearing through increased head motion oriented mainly toward the working ear. Additionally, it should be noted that sound localization was affected to a greater degree in case of moving acoustic stimuli compared to those standing still. Finally, the constant position of eyes indicates that vestibulo-ocular reflexes were activated.

In particular, Coudert et al.’s work [70] examined the ability of children and adolescents using bilateral cochlear implants (BCIs) to locate 3D sound sources compared to controls with normal hearing capacity. In an immersive virtual reality setting, head and hand movements were tracked, and precise control was exercised over the visual input and starting eye position of each subject. The analysis focused on precision in locating the source of sound at various distances and angular locations, contrasting two conditions: “head still” and “head moving.” It emerged that BCI patients were able to recognize laterality accurately but lacked spatial precision, with frequent errors of antero-posterior recognition and problems with recognizing distance. Nevertheless, it should be noted that spontaneous head movement proved to be a critical mechanism of compensation in such cases: the capability to explore the environment kinesthetically greatly diminishes localization errors.

The progress of eye tracking techniques turned out to be the reason why the saccadic reflex towards the location of the auditory stimulus can be utilized in clinical research easily. The research conducted by Eklöf et al. [71] evaluates the growth of latency for sound localization (SLL) among children with normal hearing (from 0.5 to 5.6 years old). The participants underwent an experiment based on 12 loudspeaker-screen pairs where audiovisual stimuli were provided to the participants. When there were azimuth changes in the sound source, the visual part was blocked so that only sound localization was estimated. With the help of sigmoidal modeling of eye tracking results, we could determine the latency time of sound localization which varied from 400 to 1400 ms. It is evident that the relationship between age and latency time was positive since the former was directly proportional to the decrease in latency. However, SLL did not correlate with localization accuracy, thus implying that eye movement speed is an independent parameter for evaluating auditory system development.

The purpose of Eklöf et al.’s [72] study was to test the reliability of a clinical procedure for the measurement of sound localization latency (SLL) using corneal reflection eye tracking with respect to the effect of simulated unilateral hearing loss (SUHL). For this purpose, the researchers examined ocular response latencies to an azimuth shift of a continuous tone signal using a sigmoid function to determine mean saccadic latency in a group of eight adults with normal hearing. These results show that when hearing is unimpaired, the mean latency value of sound localization is 280 ms. However, when unilateral hearing loss was simulated (30 and 43 dB HL), there was a proportional rise in latency value (370 ms and 540 ms, respectively). An extremely high correlation coefficient (R^2^ = 0.94) was identified between response time and spatial localization precision.

The effectiveness of BCIs in enhancing the accuracy of sound localization in CHL subjects was examined in Asp et al. [73], who investigated the efficacy of bone-conducted implants in the improvement of sound localization abilities in patients with unilateral or bilateral hearing impairments. In their experimental prospective study, the subjects’ capability to orient themselves horizontally was evaluated in terms of the error index (EI) when using the implant on one side compared to no hearing aid at all. Behavioral outcomes were objectively collected using the eye tracking technique. The findings showed that more than 60% of the subjects benefited from using the bone conduction implant. Moreover, in the presence of bilateral hearing impairment, there was a significant positive linear correlation between the hearing threshold level of the contralateral ear and the degree of improvement in sound localization ability.

The discrimination between ITDs in a bilateral pediatric population implanted with CI was examined by Eklöf et al. [74]. The effect of FS processing techniques was evaluated. In this study, eye tracking technique was employed to test horizontal localization by measuring JND for ITD and ILD. It is found that sensitivity to ITD can be demonstrated only in subjects equipped with a processor having a FS processing technique. However, this factor seems not to enhance sound localization performance directly. On the contrary, localization of broadband sounds was significantly related to the sensitivity to ILD. Moreover, localization of lower frequencies was harder than the one of broadband stimuli. Thus, despite the necessity of FS coding for temporalization, the intensity difference optimization remains crucial for spatial localization in pediatric implantation.

The following table presents the principal studies of auditory perception in pediatric and clinical populations carried out for hearing aids validation and sensorimotor development examination (Table 9).

A discussion of the results presented in Table 9 emphasizes the objective nature of eye tracking as one of the most effective diagnostic tools compared with conventional methods, particularly in the field of pediatrics. In contrast to behavioral and verbal tests, eye tracking provides constant measurements and does not require invasive procedures to evaluate the patient’s perceptual skills. One of the interesting points mentioned relates to compensatory motor functions, especially the movement of the head, which plays a significant role in enhancing spatial orientation in people with hearing disorders. This process reveals the possibility of adaptation of the sensorimotor system, relying on the interaction of visual, vestibular, and auditory signals. Another important element highlighted by the results is connected to the significance of new coding approaches of signals, such as Fine Structure, enabling the improvement of their perception due to a more elaborate representation of sound. Their implementation in prosthetic devices leads to the improvement of spatial orientation and, thus, to a more positive experience of patients regarding hearing.

#### 4.3.4. Spatial Perception in Interactive Environments

The first part of this section is devoted to the discussion about immersive technologies and interactive media as tools for experimental investigation of spatial hearing, where an important emphasis will be made on the importance of integration between VR, MR, etc., with multisensory perception and exploring behavior. In particular, using virtual reality and other types of environments, one can create the ecologically valid yet controlled experimental conditions that enable precise investigation of the phenomenon of auditory orienting when different kinds of visually perceived stimuli are present. One of the important features in the field concerns the effect of visual perception on the perception of auditory orientation in space. Visual perception could play an important role in this process since it might greatly affect the way visual stimuli influence the fixation distribution and searching strategy during the auditory exploration. Also, movement freedom provided by immersive virtual environments creates new motor and perceptual variables influencing vestibular–visual-auditory interaction. These technologies make it possible to study the phenomenon of auditory orienting in a realistic manner, unlike the laboratory paradigm of experiments.

The use of eye tracking in translation studies has been gaining traction since the middle of the 2000s decade, making possible the interpretation of the cognitive mechanisms associated with the linguistic process of interpreting and receiving messages. Kudla et al. [75] focus their research on video game localization, a special field because of its multimodality, that is, the emergence of the message through the interaction between images, texts, speech, sounds, and interactivity, the latter being key, because the user is no longer a passive receiver, as it happens in audiovisual translation, but a participant in the narrative, who can dramatically change their fixation patterns. This study addresses the issues associated with the methodology of research on this topic, specifically the importance of triangulating data from eye movement analysis with other sources of data. It focuses on hardware and software requirements, selection of participants and samples, and selection of segments in games, among others.

The problem of interaction between sound perception and vision has been extensively discussed in scientific works with emphasis being put on the phenomenon of the ventriloquist effect; however, there has been no investigation of the impact of the structure of the space on sound localization. Using an innovative paradigm that included virtual reality, real sounds, and real-time cinema tracking, Valzolgher et al. [76] conducted an experiment aimed at studying how a minimalistic visual setting affects spatial hearing. Participants with normal hearing had two test settings—the first one represented the gray neutral background, and the second one was a perspective grid without any information on the place of origin of the sound. Results have shown that a simple visual structure can positively affect sound localization by enhancing pointing precision and improving the speed of the eye response to sound stimulation.

Despite the importance of spatial hearing, conventional audiological test batteries often provide limited data on the localization and tracking of dynamic stimuli. To address this gap, Fischer et al. [77] implemented a dynamic free-field experimental setup, evaluating twelve normal-hearing subjects. Participants were asked to indicate the location of sounds using two modalities: a touchpad or eye tracking. The analysis reveals that discrimination and tracking performance is superior in the frontal azimuth compared to the posterior azimuth, with a positive exception along the posterior midline. As hypothesized, free head movement optimized tracking capabilities. However, using eye tracking as a feedback method generated higher tracking errors than using haptic feedback. Finally, the observed correlation between static and dynamic assessments supports the “snapshot theory” of auditory motion perception, suggesting that the auditory system processes motion as a sequence of discrete positions.

Table 10 presents an overview of the most relevant studies conducted in virtual reality environments and gaming contexts, focusing on the analysis of multisensory dynamics in sound localization. Experimental approaches, immersive configurations, and key findings are summarized, highlighting the interaction between visual and auditory stimuli and exploratory behavior.

The discussion of the results presented in Table 10 shows how important the use of immersive technology, especially in the form of virtual reality and cinematic tracking systems, is in the process of increasing the comprehension of the mechanisms of sound localization under controlled ecological conditions. The use of these instruments allows separating all the factors that have their contribution to perceptual processes by dividing the processes into the visual, auditory, and motor elements of the activity. In particular, one of the elements that become apparent is the impact made by the visual frame, which serves as a constant spatial frame of reference and affects significantly the oculomotor exploration patterns. Moreover, the combination of visual and acoustic stimuli, either in the coherent or competing way, can have an effect on the time required for orientation and the precision of the response itself. Another important feature provided by the interactivity specific for digital media is the alteration of attention and perceptual processes.

Finally, the literature converges on the idea that eye-tracking information—such as fixation distributions, gaze patterns, and scan path dynamics—can effectively complement acoustic features in sound event detection and classification tasks, improving both model robustness and interpretability, while also revealing a general trend toward multimodal learning approaches that better capture human perceptual relevance in complex auditory scenes.

### 4.4. Eye Tracking-Based Acoustic Sensing and Multimodal Systems

In this part, eye tracking can be introduced as one of the fundamental techniques for studying behavioral and cognitive processes, as it provides a reliable tool for analyzing how people process information about their visual environment. Modern advancements in computer vision, artificial intelligence, and miniaturization of sensors make possible creating more sophisticated systems which are able to operate in real time and work with different types of inputs simultaneously. Moreover, being incorporated into VR/AR environments makes them even more applicable for various purposes, including education, communication, and research of human behavior. In this way, recent developments in eye tracking technologies can be seen as a transition to some alternative approaches that combine acoustic sensing and multimodal processing. The employment of ultrasonic or acoustic waves enables estimation of the direction of the gaze, as well as other physiological indicators of human activity, without the need to rely on illumination and providing additional advantages in terms of privacy and power consumption. Moreover, the fusion of acoustic data with eye tracking and other sensors results in the emergence of multimodal systems which can analyze the actions of users, recognize gestures or facial expressions, and facilitate interactions with smart devices. This integrated approach allows not only the capture of individual signals, but also the exploitation of relationships between different sensory modalities. Overall, this research reflects a transition from the use of eye tracking as an analytical tool towards its integration as an active component in intelligent systems, leading to portable, continuous and contextually adaptive solutions with applications in human–computer interaction, physiological monitoring and advanced interactive environments.

#### 4.4.1. Acoustic-Based Eye Tracking and Physiological Monitoring Systems

In this subsection, we will discuss the eye trackers based on acoustic and ultrasonic technologies, which utilize inaudible signal trans-mission and echo detection for precise determination of eye-gaze direction and eye activity without the use of cameras, avoiding privacy issues, reducing power consumption, and increasing system robustness in different lighting environments. In addition to determining eye gaze direction, these technologies provide information about other aspects like blinking and cognitive load by capturing acoustic echoes, and this section will also address simulation techniques, which involve generating artificial data to improve model training and tuning.

The article of Li et al. [13] present GazeTrak, an acoustic-based eye tracking system integrated into glasses, designed to estimate the direction of gaze without the use of cameras. The method employs a loudspeaker and four microphones that emit inaudible sound towards the eyes and process the reflected sound waves using a deep learning model to continuously infer the position of the gaze. The experimental study involved twenty participants. The results show an accuracy of 3.6 degrees within the same session and 4.9 degrees across sessions, with a refresh rate of 83.3 hertz. Furthermore, implementation on a low-power microcontroller reduces energy consumption whilst maintaining high performance.

The study by Sun et al. [78] present a portable ultrasound-based eye tracking technique designed to overcome the limitations of traditional methods in terms of privacy, comfort and resource consumption. The method employs arrays of microelectromechanical ultrasonic transducers integrated into glasses, which transmit and receive echo signals to estimate eye movement using the time-of-flight principle. The experimental study was conducted with volunteers; neither the sample size nor the location of the study is specified. The results show that the system enables real-time tracking of the direction and rotation of the eyeball with high precision, whilst also highlighting its small size and light weight, which supports its potential in portable applications such as virtual reality.

Liang et al. [79] have developed an ultrasound-based blink monitoring system with the aim of providing a non-invasive, safe and comfortable alternative to traditional methods. The method is based on a dual-electrode piezoelectric microelectromechanical transducer integrated into a pair of glasses, which uses time-of-flight measurements of ultrasonic signals alongside unsupervised machine learning techniques to identify blinking states. Neither the sample size nor the location of the study is specified. The results show that the system enables the detection of blinking in real time with high reliability, whilst also highlighting its compatibility with standard manufacturing processes and its potential for continuous monitoring applications.

The research of Lu et al. [80] propose an ultrasound-based simulation platform designed to generate realistic data for eye tracking on wearable devices. The method is based on a synthetic face and eye model that allows for variation in gaze direction, eyelid opening and device movement; this data is processed using machine learning algorithms to estimate gaze and system drift simultaneously. Neither the sample size nor the location of the study is specified, as this is a simulated environment. The results show a mean squared error of 0.085 degrees without displacement and 0.756 degrees with displacement, demonstrating the platform’s usefulness for optimizing the design of eye tracking systems.

Table 11 summarizes studies on acoustic-based eye tracking systems and integrated physiological monitoring. These multimodal approaches combine eye, sound, and biometric data to analyze attention, perception, and cognitive states, highlighting new opportunities for advanced applications in science and technology.

The results summarized in Table 11 highlight the rapid advancement and growing reliability of acoustic-based eye tracking and integrated physiological monitoring systems. Reported accuracies of 3.6° within sessions and 4.9° across sessions demonstrate a promising level of consistency, particularly considering the challenges associated with cross-session variability. The achieved value of 83.3 Hz refresh rate again shows its appropriateness for real-time application as it makes it possible to register dynamic eye movements at sufficient resolution while maintaining a low level of power consumption—another indispensable quality of the technology that makes it suitable for use in wearable devices. Accurate registration of eye direction and rotations testifies to the viability of the acoustic-based approach as an efficient light-weight method in comparison with optical approaches, which is especially important when there is a possibility of ocular or light obstruction. Reliable real-time detection of blinking also speaks to the possibility of registering such physiological processes using acoustics as well. Low error rates, specifically the value of mean squared error of 0.085°, speak to the high potential of the technology in terms of accuracy of eye movement recognition. While the value of mean square error is considerably higher for displacement (0.756°), it provides us with valuable information that is useful for further calibrating our system.

#### 4.4.2. Acoustic Sensing for Facial Interaction and Behavioral Analysis in Wearables

This sub-section highlights studies related to the application of sound sensors in wearable gadgets, mainly smart glasses, for the detection and decoding of facial expressions and activities of the users, thereby facilitating novel means of interaction between humans and computers. The studies have shown that ultrasonic waves can sense minute movements of the skin and facial muscles, and thus expressions or actions can be recognized without touching or using any cameras. Building on this capability, applications are being developed ranging from eye-gesture-based interfaces to facial expression recognition systems and the monitoring of behaviors such as food intake. Furthermore, the use of these signals for biometric purposes is being explored, enabling users to be identified quickly and in a non-intrusive manner. Overall, this subsection reflects a shift towards natural, continuous and contextually adaptive interfaces, based on the interpretation of physiologically derived signals captured acoustically.

Sun et al. [81] propose an interaction system for smart glasses called EyeGesener, based on eye gestures detected using acoustic signals, with the aim of enabling hands-free control and preventing unintended activations. The method uses integrated speakers and microphones that capture eye movements via acoustic signals processed using advanced channel estimation and machine learning techniques, including adversarial training to generalize across users. The experimental study was conducted with sixteen participants. The results indicate that the system recognizes eight eye gestures with an average F1 score of 0.93 and a false alarm rate of 0.03, whilst also demonstrating high usability in a real-time interactive application.

The research of Xie et al. [82] propose an acoustics-based system for recognizing upper facial actions designed for smart glasses, to enable more natural, hands-free interaction. The method uses built-in speakers and microphones that capture subtle variations in facial expressions via acoustic signals, which are then processed using time-frequency analysis techniques and deep learning models to classify six facial actions. The experimental study was conducted with twenty-six participants. The results show that the system achieves an average F1 score of 0.92 in identifying these actions, demonstrating its effectiveness as an alternative to traditional interaction methods.

The study of Mahmud et al. [83] introduces MunchSonic, an active artificial intelligence-based system integrated into glasses to monitor eating actions with the aim of identifying behaviors related to food intake. The method uses ultrasonic acoustic signals emitted from the frame of the glasses; the echoes from these signals capture movements of the mouth, jaw and limbs, which are then processed using deep learning models to classify six types of actions. The study was conducted with twelve participants in natural settings. The results show an average F1 score of 0.935 in independent user evaluation, with a temporal resolution of two seconds, further demonstrating its effectiveness in detecting eating episodes and the frequency of food intake.

Li et al. [84] present SonicID, an authentication system for smart glasses designed to identify users quickly and in a non-intrusive manner. The method uses ultrasonic signals to scan the face and extract biometric features, which are processed using a deep learning model based on a binary classification architecture. The experimental study was conducted with forty participants. The results show a true positive rate of 97.4%, a false positive rate of 4.3% and a balanced accuracy of 96.6%, using one minute of training data per user, whilst maintaining stable performance across different sessions.

In another paper, Li et al. [85] present an acoustic detection system integrated into glasses for the continuous tracking of facial expressions, with the aim of capturing facial movements discreetly. The system is called EyeEcho. The method uses pairs of speakers and microphones that emit inaudible signals towards the face and analyze skin deformations using machine learning models to estimate facial movements. In the experiment, there were twelve subjects used during the control trials, while ten others participated in the natural condition’s trials, but the site of the experiment is unknown. High levels of accuracy achieved within only four minutes of training data are observed, providing the same level of effectiveness across several different daily situations.

Table 12 shows a summary of research on the utilization of acoustic sensing through wearable devices in order to analyze facial interactions and human behavior. In this case, acoustic signals are being utilized in order to identify movements of the face, expressions, and other actions performed by individuals.

The studies by Mahmud et al. [83] (MunchSonic), Li et al. [84] (SonicID), and Li et al. [85] (EyeEcho) are grouped within the section Acoustic Sensing for Facial Interaction and Behavioral Analysis in Wearables due to their shared methodological and conceptual foundation. In particular, all three contributions rely on wearable devices, specifically smart glasses, equipped with acoustic transducers and microphones to enable non-invasive sensing of facial or oral-region dynamics. Despite addressing different application scenarios, they consistently exploit ultrasonic or inaudible acoustic signals to capture human-related information through reflected or modulated sound waves. MunchSonic focuses on the detection of eating behaviors through jaw and mouth activity analysis, SonicID targets user authentication via acoustic-based facial biometric signatures, and EyeEcho enables continuous tracking of facial expressions by estimating subtle skin deformations. Collectively, these studies demonstrate how acoustic sensing embedded in wearable platforms can support behavioral monitoring, identification, and expression analysis, thus contributing to the broader theme of multimodal, contactless human–computer interaction systems.

The results reported in Table 12 demonstrate the effectiveness of acoustic sensing approaches for facial interaction and behavioral analysis in wearable systems. Acoustic methods, which rely on speakers and microphones, along with channel estimation and adversarial machine learning, provide excellent results because they are able to recognize eight different eye gestures with an F1 score of 0.93, while producing a relatively low false alarm rate of 0.03. At the same time, solutions using time-frequency analysis along with deep learning models are capable of recognizing multiple actions on the face, thus achieving an F1 score of 0.92. This proves the effectiveness of this technology in hands-free interaction scenarios. Additionally, ultrasonic sensing provides good results because it allows one to achieve an F1 score of 0.935 while analyzing eating behavior and frequency. Also, biometric-related approaches that use ultrasonic facial scanning have been proven to be effective as they achieve a true positive rate of 97.4% and balanced accuracy of 96.6%. What is particularly interesting is the fact that some systems are able to achieve excellent results in just four minutes of training, but at the same time, they are able to maintain consistent re-real-time performance.

#### 4.4.3. Multimodal Systems Integrating Eye Tracking and Acoustic Information

This sub-section presents work involving eye tracking combined with acoustic and other sensory information in order to better understand the environment and interact with sophisticated systems. Multimodality is an unmistakable feature of all these approaches, combining vision and hearing and sometimes even the behavioral input of the users to gain a more comprehensive picture of the context and make better decisions accordingly.

Through the combination of these sources of data, several types of applications have been developed including automatic control of gadgets according to gaze tracking and acoustic activity; measuring sound localization skills through responses to visual stimuli; enhancing speech recognition technologies by interpreting user intentions and other similar purposes such as monitoring driver attention or sports performance. Overall, this subsection highlights the value of sensor fusion for developing more robust, context-aware, and intelligent multimodal interactive systems.

Kotus et al. [86] present an innovative system that integrates eye tracking and vector acoustic sensors to guide mobile cameras with orientation and zoom control. The aim of the study is to optimize automatic camera pointing by combining visual attention and the localization of sound events. Image processing and camera control algorithms are employed, alongside two modes of operation: one in which the audio aligns with the visual orientation, and another in which the sound directs the camera’s movement. Five PhD students participated in the study. The results indicate an improvement in the detection and tracking of relevant events, as well as in user interaction.

Asp et al. [87] have developed an objective, efficient and reliable method for assessing sound localization accuracy from six months of age. The study is based on recording pupillary position via eye tracking using corneal reflection in response to spatially distributed auditory and visual stimuli. They used the Smart Eye Pro system (not developed by them). Twelve children aged between 29 and 157 weeks and eight adults with normal hearing took part in an audiological room equipped with multiple sound sources. The results show that the measurement takes just a few minutes, with high accuracy in adults and lower accuracy in children, although with progressive improvement with age. It is concluded that the method allows for the objective assessment of sound localization and has potential as a clinical tool in early-stage populations.

Ohneiser et al. [88] analyze voice recognition systems for air traffic controllers through the use of eye tracking and mouse data. The aim is to improve the accuracy of command prediction and reduce the intrusiveness of the confirmation process. Techniques for analyzing visual focus and user interaction are employed to infer intentions and validate results. The study is based on a single experimental case involving two participants. The results demonstrate significantly improved accuracy of predictions and decrease in errors. The fact of the possibility of using visual verification for replacing manual labor processes is proven.

Langner et al. [89] have developed a method aimed at continuous monitoring of a driver’s degree of attention in traffic conditions. The idea behind the project consists of detecting possible distractions and warning the driver about unnoticed dangers. The technique involves computer vision-based recognition of traffic light and obstacles, estimation of attention and head position by means of eye tracking, and visual and sound prompts provided through human machine interface. The implementation of the algorithm occurs in an autonomous car functioning as an experimental test bed. The results show that the proposed method allows detecting driver’s distraction and helping him by issuing warnings.

Seong et al. [90] have created a dataset for badminton analysis, which could be used to improve personalized training systems through providing feedback on athlete’s performance in real time. Different sensors were used in order to collect information, among them there are eye tracker, body movement sensor, muscle signals and foot pressure, along with video records and detailed shots analysis. The sample comprises twenty-five players of varying skill levels, with a total of 7763 movements recorded, and data collection takes place in an experimental sports training environment, without specifying a particular location. The results demonstrate the usefulness of the dataset for analyzing the biomechanics of the sporting movement and validating machine learning models applied to advanced training.

Table 13 summarizes studies on multimodal systems integrating eye tracking and acoustic information. These approaches combine visual attention data with auditory signals to enhance the analysis of perception, behavior, and interaction, highlighting advances in sensor fusion, data processing techniques, and applications in complex, real-world and human–machine interaction scenarios.

Results presented in Table 13 emphasize the substantial advantages offered by multimodal systems combining eye tracking with acoustic data. The common conclusion made by researchers is that combining complementary cues contributes to better identification and tracking of important events. The incorporation of eye tracking into multimodal interaction provides increased sensitivity in terms of event recognition, which leads to improved interaction and multimodal control strategies. Clinically, multimodal systems offer quick and accurate measurements of adults with high precision, while their performance with children, being still worse than in adults, demonstrates significant improvements. There is, therefore, much promise for pediatric applications as well as diagnosis due to further improvement in the model design. In addition, the combination of visual feedback with acoustic cues helps avoid manual data input, increasing speed and reducing errors. In safety-critical applications, the use of multimodal sensing techniques contributes to detecting signs of driver distraction and issuing appropriate alerts, leading to increased road safety. In addition to monitoring functions, multimodal frameworks are effective in biomechanics analysis due to collecting of behavioral and sensory information at the same time. Large amounts of annotated data sets, consisting of thousands of performed actions, help develop more sophisticated algorithms and personalized training schemes.

#### 4.4.4. Robotic Audition and Audio–Visual Integration Systems

The second part focuses on robots that utilize an auditory and visual perception system for interacting automatically in a changing environment with multiple sound sources. This section emphasizes the need for active listening where the robot adapts its orientation and actions to enhance the process of perception. In such an instance, there is a trend for developing systems capable of locating, separating, and recognizing multiple sound sources at the same time through auditory and visual tracking, directional filtering, and sophisticated recognition methods. Movement of the robot enhances the perception process, creating processes similar to those in the fovea. Taken together, these systems represent a step towards robots with sensory capabilities closer to those of humans, capable of functioning in complex real-world environments.

Trifa et al. [91] address human–robot interaction through the integration of multisensory information, with the aim of improving the localization of sound sources and the robot’s response to environmental stimuli. The methodology is based on the combination of acoustic and visual data, as well as on the comparison of various sound localization algorithms for real-time use. In addition, a distributed control framework is implemented that allows the integration of other modalities such as speech recognition and visual tracking. The system is applied to a humanoid robot, without specifying the sample size or the location of the study. The results show a more natural and effective response to stimuli, improving interaction.

The authors Nakadai et al. [92] propose an active robot auditory system aimed at improving the performance of sound localization, separation, and identification in environments containing simultaneously uttered speech in noisy conditions. This technique is composed of three subcomponents that are real-time tracking of individuals using a combined approach of audio–visual fusion, adaptive directional filtering for sound separation, and multiple acoustic model-based recognition system. Additionally, the technique includes movement control that is based on directing movement towards the source of sound based on the concept of the auditory fovea effect. Improvements in sound localization, sound source separation, and speech recognition have been achieved.

Table 14 provides examples of research works related to robotic audition and audio–visual systems. Such techniques incorporate acoustic sensing capabilities with visual sensing in order to facilitate environmental interpretation, sound source localization, and communication between humans and robots.

The analysis conducted above and summarized in Table 14 clearly shows the high efficiency of the use of robotic audition and audio–visual integration techniques to improve the perceptual skills of robots. The first important result obtained during this research is associated with better sound source localization and the increase in robot reactivity due to the combination of auditory and visual signals. Through the application of speech recognition combined with visual tracking and distributed control approaches, much more advanced and more realistic human–robot interactions become possible. The second important innovation related to audio–visual integration lies in its association with adaptive directional filtration and multimodal acoustic models. This allows for the creation of an improved signal separation system and better speech recognition. It should be stressed that the inclusion of motion-based active audition mechanisms, referred to as an “auditory fovea,” makes it possible for robots to orient towards interesting sound sources, making localization more efficient and precise. This multimodal synergy is especially valuable in real-world scenarios where noise, occlusions, and environmental variability challenge traditional unimodal systems, paving the way for more autonomous and adaptive robotic platforms.

Finally, the reviewed works indicate a clear shift from eye tracking as a standalone observational tool toward its integration within multimodal acoustic–visual sensing systems, where ocular metrics and oculomotor behavior are jointly exploited with acoustic signals to enable more robust, context-aware, and real-time human–machine interaction, although challenges remain in achieving seamless sensor fusion and scalable deployment across heterogeneous environments.

### 4.5. Eye Tracking in Soundscape and Perceptual Acoustic Studies

Recent research on soundscapes has increasingly adopted an integrative perspective in which sound is treated as a core component of environmental experience rather than a purely physical phenomenon. Acoustic environments shape human perception through cognitive and affective processes, influencing how spaces are interpreted and experienced. Within this framework, particular attention has been given to contrasts between natural and artificial sounds and their effects on outcomes such as well-being, attentional engagement, and subjective environmental evaluation. In parallel, eye tracking has become a key methodological tool for objectively examining visual attention and perceptual mechanisms. This approach offers more continuous and finely detailed analysis of eye movement compared to self-reporting methods, allowing for a more detailed study of the processes of fixation and visual exploration strategy used by subjects to perceive the visual world around them. The combination of these methodologies has allowed a considerable amount of research related to audio–visual perception to be conducted, which analyzes auditory cues in connection with visual attention dynamics and scene perception.

The result of such research can allow for identification of interactions between auditory and visual perceptions, which can have an effect on the state of restoration and emotional and cognitive responses of the subject.

#### 4.5.1. Soundscapes and Psychological Restoration in Natural and Urban Environments

This section brings together studies that analyses the role of soundscapes in improving well-being, emotional regulation and cognitive restoration, incorporating eye tracking to examine visual attention. The research is conducted in natural, educational and urban settings, using audiovisual stimuli or real-world environments. The findings agree that natural sounds (such as water, wind or birds) have a positive effect on emotional state, reduce mental fatigue and promote psychological recovery. Furthermore, these stimuli direct visual attention towards natural elements, reinforcing their restorative impact. Evidence suggests that the soundscape does not act in isolation, but rather in interaction with other sensory stimuli, which is key to the design of healthy environments.

The study by Li et al. [15] explores the relationship between visual attention and tourists’ emotional responses to images of destinations, additionally considering the influence of perceived stress. To this end, eye tracking techniques were used in combination with visual, auditory and textual stimuli applied to images of natural and urban environments. The inclusion of auditory stimuli distinguishes this study from similar research using virtual reality [93,94,95]. The sample included participants with varying levels of stress, allowing for a comparison of their responses. The results show that auditory stimuli increase attention towards natural elements and generate positive emotions, whilst textual stimuli direct attention towards built elements, although in excess, they can prove distracting. Furthermore, attention to natural environments is associated with more positive emotional responses, particularly in individuals with lower stress levels.

Fei et al. [96] investigate how the soundscape in the public spaces of primary schools’ influences children’s perception, cognition and emotions. To this end, they developed an experiment based on virtual audiovisual scenes featuring seven types of soundscapes and three spatial variables, applying eye tracking techniques and physiological measurements (electrodermal activity and heart rate) to a sample of 101 schoolchildren. The results show that natural sounds enhance environmental perception and cognitive recovery, with birdsong standing out as the most effective one. Sounds from the school environment, such as laughter, improve attention and emotional state, whilst traffic noise increases mental fatigue. Furthermore, the effects vary depending on the type of space, suggesting the need to design differentiated soundscapes in educational settings.

Liang et al. [16] analyze how the soundscape of a university campus influences students’ emotions and well-being, using the Qishan campus of Fuzhou University as a case study. The study evaluates four functional areas (academic, residential, recreational and administrative) using techniques such as electroencephalography, eye tracking, acoustic measurements and questionnaires. 30 participants took part in the study. A repeated measures analysis of variance was applied to examine the effects of different soundscapes. The results show that natural sounds, such as wind or water, improve emotional state and promote psychological recovery, whilst artificial noises, such as traffic or construction, generate negative effects. Furthermore, the subjective perception of restoration is positively related to acoustic comfort and positive emotions.

The study by Kou et al. [97] examines how the various sensory components of the natural environment influence the psychological recovery of people experiencing stress and mental fatigue, considering the reduced contact with the outside world during the pandemic. To this end, they employ a mixed-methods approach combining questionnaires, in-depth interviews, real-time location data, acoustic measurements and eye tracking techniques. The survey was conducted in the Huangshan Scenic Area, yielding 405 valid responses. The results show that visual, olfactory, and tactile stimuli directly influence the restoration of attention, whilst the soundscape acts indirectly by generating positive emotions. Furthermore, those who reduced their exposure to the outdoors the most derive greater benefits from multisensory interaction with nature.

Zhu et al. [98] analyze the restorative effects of audiovisual environments in bamboo forests on university students. The study was conducted in the Bamboo Sea of southern Sichuan, where images of four types of spaces were collected and combined with different soundscapes. 32 current college students participated in the study. Perceptual responses were assessed using eye tracking experiments. The results show that recreational and ornamental spaces have greater visual restorative capacity, whilst natural sounds such as wind, water or birds improve subjective perception and reduce indicators of fatigue. Furthermore, sound influences visual attention, enhancing focus on natural elements.

Ren et al. [99] analyze how soundscapes influence the assessment of rural landscapes in terms of perceived visual quality, tranquility, and preference. The study aims to examine the effect of sound on the assessment of different types of rural landscape and its usefulness for environmental assessment and landscape design. Audiovisual experiments and eye tracking tests were carried out in the laboratory, using images and sounds from rural villages in China, not from a single global sample, but featuring various types of landscapes such as fields, water, paths, and dwellings. Twenty students took part in the study. The results show that natural or musical sounds significantly improve ratings compared to silence or artificial sounds, particularly in terms of tranquility and preference, whilst sound elements also alter visual attention as measured by eye tracking, highlighting their influence on the perception of landscape elements.

Weng et al. [100] evaluate the effectiveness of Shinrin-yoku (a nature-based practice that involves the five senses within a forest environment) in contexts of increasing urbanization. The study analyses visual attention, perception of the acoustic environment, and physiological and psychological responses in forty-one participants over three days in various forest settings within Wuyishan National Park. Techniques including eye tracking, questionnaires, measurements of heart rate variability and skin conductance, alongside psychological scales, were employed. The results show that natural and cultural stimuli positively influence physiological regulation, reduce stress and promote positive emotional states. It is concluded that exposure to natural environments contributes significantly to general well-being.

Table 15 summarizes studies investigating soundscapes and psychological restoration in natural and urban environments. These works explore how acoustic environments influence stress recovery, attention restoration, and emotional well-being, highlighting differences between natural and urban settings. The review emphasizes methodological approaches, perceptual metrics, and key findings in environmental sound perception research.

The results summarized in Table 15 consistently demonstrate the strong restorative role of soundscapes in both natural and urban environments. Overall, auditory stimulation from nature tends to increase attention towards natural aspects and evokes more positive emotions than man-made stimuli, especially when stress levels are low. Man-made sound, such as traffic noise, often correlates with tiredness and uncomfortable emotions. On the other hand, bird sounds have proven to be the most effective for achieving a state of cognitive and affective restoration. The spatial context of sound perception is also highly significant in this regard. The acoustic setting in recreational or natural environments was found to promote restoration, whereas artificial environments were less effective in this case. In addition, the results indicate that soundscapes impact visual attention through emotional mechanisms. Even though visual stimuli were shown to have a direct effect on attention restoration, auditory stimuli seem to help restore cognitive and emotional well-being and thus impact perceptual processes indirectly. Moreover, there is evidence that combining sounds from both natural and cultural contexts helps to lower stress levels. Overall, these results confirm that sound is a key determinant of environmental perception, capable of significantly modulating attention, emotion, and restorative processes in both natural and urban contexts.

#### 4.5.2. The Influence of the Soundscape on Visual Attention and Multisensory Perception

This section focuses on how soundscapes influence the distribution of visual attention and the multisensory perception of the environment, using eye tracking as the primary analytical tool. Studies include immersive environments, virtual reality, and controlled experimental scenarios. The findings show that sound can modulate the direction, intensity, and dispersion of visual attention, especially when combined with complex visual stimuli. Elements such as spatial audio and audiovisual congruence enhance immersion and facilitate the interpretation of the environment. Furthermore, it is demonstrated that the perception of the soundscape depends on the integration of sensory modalities, making it a dynamic process influenced by context.

Oberman et al. [101] study the influence of visual information on the perception of sound environments. The aim was to assess whether participants’ perceptions of the soundscape and their behavior could be explained by visual stimuli. A laboratory study was conducted using questionnaires and eye tracking, employing 360-degree videos and spatial recordings of 27 urban spaces, presented via virtual reality. The results revealed significant differences between sound-guided and vision-guided perceptions, as well as variable interactions that alter the direction, intensity and dispersion of responses. It is concluded that the perception of the sound environment depends on multiple interrelated contextual factors; therefore, it cannot be explained by a single environmental attribute, and a comprehensive approach is required for its planning.

Hirway et al. [102] analyze how audio settings influence the user experience and visual attention in 360-degree immersive videos. To this end, they compare spatial and non-spatial audio using a methodology that combines subjective evaluations with eye tracking, head-orientation analysis and physiological measurements (including heart rate and pupil dilation). Responses from 73 participants, distributed across the study’s three conditions (no sound, first-order, second-order and stereo sound). Preliminary results indicate that spatial audio increases the sense of immersion and directs visual attention more effectively. Furthermore, the findings suggest practical applications in content creation and transmission optimization, enhancing the experience in environments with bandwidth limitations.

Wright et al. [103] investigate how multimodal sensory substitution influences object localization when visual and auditory information do not match. The aim of the study is to analyze whether the integration of natural vision and sound generated from vision improves the detection of a hidden auditory target. A laboratory experiment involving eye tracking was conducted with a sample of participants of unspecified size, in which subjects were required to localize an object present only in the auditory channel within complex visual scenes. Forty-eight students took part in the study. The results show that performance improves when visual background information is present, as it helps to generate predictions about the sound environment. Furthermore, eye tracking reveals that eye movements are directed towards the location of the auditory target even when responses do not match, demonstrating the potential of multisensory integration to guide attention in complex tasks.

Kothinti et al. [104] investigated auditory salience, defined as the ability of certain sounds to capture involuntary attention. To this end, they used an online platform incorporating a dichotic listening paradigm and eye tracking techniques, employing natural auditory stimuli. They validated the collection of responses via crowdsourcing by comparing the results with data obtained in the laboratory. The sample included a large number of participants who assessed various sound scenes. The findings showed that extending behavioral measures to different auditory environments and increasing the number of subjects allows for a better understanding of auditory salience, demonstrating that low-level acoustic attributes do not adequately capture the richness of natural sounds.

Table 16 summarizes studies on the influence of soundscapes on visual attention and multisensory perception. These works investigate how auditory environments shape gaze behavior, attentional allocation, and perceptual integration across senses. The review highlights experimental paradigms, key findings, and the role of acoustic context in modulating visual and cognitive processing.

The results presented in Table 16 indicate a high level of context-dependent interaction between auditory and visual modes in influencing multimodal perception. First, it is worth noting that the presence of visual cues affects the perception of soundscape, resulting in significant differences in assessments performed with respect to the same environment depending on whether it was evaluated through visual or auditory means. Second, visual cues play an essential role in influencing immersion, allowing for more effective guidance of the user’s visual focus. As such, it appears to be applicable in designing immersive media and facilitating bandwidth-efficient rendering of virtual environments. Third, there is evidence that auditory targets benefit from visual context cues in terms of localization. Although there is no direct correlation in overt behavior between sound and vision, it seems like the two modalities are highly integrated in human cognitive processes, at least implicitly. Fourth, there is proof that auditory saliency depends on complicated acoustic structures, not only basic low-level properties of stimuli. Finally, large-scale behavioral studies prove that natural sound complexity is better accounted for in a multimodal environment, as opposed to controlled laboratory settings.

#### 4.5.3. Soundscape, Cognitive Load, and Human Behavior

This section includes studies that examine the impact of the soundscape on cognitive processes, mental workload, and decision-making, integrating eye tracking measures and other psychophysiological indicators. The findings show that certain acoustic environments, particularly those involving noise or irrelevant stimuli, can increase cognitive load and mental effort, even without affecting observable performance. On the other hand, sound can also influence behavior, such as decision-making, by modulating visual attention patterns. These studies highlight the importance of considering the soundscape in functional contexts such as workplaces and consumer environments.

Grenzebach et al. [105] investigate how noise affects cognitive performance beyond its auditory effects. The aim was to assess how irrelevant auditory stimuli during cognitive tasks increase mental load. Various types of noise were analyzed, ranging from speech to irrelevant sounds, tone sequences or auditory stressors, alongside a variety of cognitive tasks, ranging from basic psychological assessments to real-world simulations. Psychophysiological indicators such as pupillometry, eye tracking, brain activity, cardiovascular and endocrinological markers, as well as behavioral markers, were used to measure cognitive load. The results show that even when observable performance remains unchanged, noise can significantly increase mental effort, highlighting the need for a comprehensive approach to prevent adverse health effects in workplace environments.

Peng-Li et al. [106] investigated how personalized soundtracks influence food choices and eye movements among consumers from different cultures. The study was conducted in a laboratory setting with 215 participants from China (114) and Denmark (101). Based on a previous study involving 396 people, two types of music were designed: one associated with healthy food choices and the other linked to unhealthy foods. Participants were assigned to listen to one of the two conditions whilst performing a food-choice task with eye tracking. The results showed that the ‘healthy’ music favored the selection of healthier foods and increased the duration and number of visual fixations on them. Statistical analysis revealed that the effect of music on choice was partially mediated by eye tracking patterns, indicating that sound can influence attention and food-related decision-making.

Table 17 summarizes studies investigating the relationship between soundscape, cognitive load, and human behavior. These works explore how acoustic environments influence mental workload, attention, and decision-making processes, often integrating behavioral and physiological measures. The review highlights key findings on the cognitive and behavioral effects of different soundscapes in real-world and controlled settings.

The results summarized in Table 17 highlight the subtle but significant influence of soundscapes on cognitive load and human behavior. A first key finding is that irrelevant or background noise can increase cognitive load and mental effort even when no measurable decline in task performance is observed. The disconnect between the effects on performance and the internal cognitive effort involved in these tasks is especially important because it implies that current measures of performance fail to capture the full extent of the influence of acoustic conditions on behavior. Within the realm of the workplace, this unmeasured cognitive effort might lead to fatigue and decreased well-being and efficiency over time, which makes it clear that acoustic design needs to take into account more than just performance and productivity. The second interesting finding of the study reveals the influence of sound on decision-making processes. Specifically, “healthy” music has been shown to increase the selection of healthy foods, as well as direct the gaze towards such foods. Eye tracking experiments have revealed that eye movement partially mediates this relationship, indicating that the sound stimulus influences behavior through its effects on visual search strategies.

In conclusion, the reviewed studies suggest that eye-tracking metrics, particularly fixation patterns and visual exploration strategies, provide complementary evidence for understanding how acoustic environments shape perceptual and affective responses, highlighting consistent cross-modal interactions between auditory and visual processing that underpin soundscape perception, attentional engagement, and restorative experience, while also pointing to the need for more integrated experimental frameworks to jointly model audio–visual perception in real-world settings.

## 5. Discussion

The reviewed papers were classified by their topic areas as follows. Four major research lines were identified to describe the trends in applying eye tracking technologies in the domain of acoustics. These research lines have been selected to facilitate understanding of the heterogenous nature of literature sources under analysis. First, sound source localization and identification [107,108] involve investigation of human ability to spatially orient themselves in the space with regard to auditory stimuli using eye behavior as the tool to reveal how concurrent sounds are localized. Second, sound event detection [109,110] and classification use eye tracking information together with computational techniques aimed at developing algorithms for automatic recognition and classification of acoustic events. Third, acoustic sensing and multimodal systems are concerned with the transition from separate techniques to an integrated approach whereby eye movements are combined with acoustic and other types of information about environment. Fourth, soundscape [111,112] and perceptual acoustic studies examine the interaction between visual and auditory stimuli in perceiving acoustic events and environments.

The following discussion presents a unified view of the empirical data obtained from the fifteen results tables, forming a coherent framework that explains the role of eye tracking in acoustic research. An important finding from the reviewed literature is the strong convergence among the neuroscientific approach to multisensory perception, the development of modern signal processing methods, and the clinical/technological applications. Unlike the previous conception that considered vision and hearing as two separate sensory channels, eye movements and sound perception are seen as parts of one system characterized by a complex interplay between its components. Feedback mechanisms, through which the interaction takes place, affect perception, attention, and behavior. In addition, the perception of sounds is an adaptive rather than linear process.

An important consideration in multisensory perception is the bidirectional nature of interactions between different modalities. While the oculomotor system used to be viewed as a motor controller responsible for performing visual tasks, it now appears that it participates actively in building auditory space representations. Auditory information, in turn, is a potent stimulus driving visual exploration. In complex environments, the use of auditory information allows the identification of relevant spatial targets regardless of any visible stimulus that may help in achieving such a purpose. In this sense, human voice or binaural rendering through HRTF shows a special ability to capture attention and to increase realism perception. Such findings point to the role played by auditory stimuli as attention-capturing elements but also as organizational elements in perceptive experience.

The link between auditory information and gaze behavior gains importance in situations where ecological validity is present, as in situations involving multiple speakers. In this context, the synchronization between the rhythms of the voice and the movements of the eyes serves to allocate efficiently cognitive resources that will allow target selection and intelligibility of the information being perceived. In this respect, it is important to point out the existence of the cocktail party effect, but extending its validity by focusing on the contributions made by oculomotor dynamics.

One of the most important technological advances is the development from uni-modal to multi-modal systems. By integrating eye tracking together with state-of-the-art signal processing of acoustics, we get the ability to build intelligent systems that can accurately interpret the surrounding environment in real time. The machine learning and especially deep learning play an essential role in the realization of this potential. Through the usage of heterogeneous information sources, like gaze direction, head orientation, and acoustic features, intelligent systems demonstrate outstanding accuracy in performing tasks like sound localization, gesture recognition, and attention determination.

One of the most creative directions for development is represented by eye tracking through acoustic means. Traditional optical systems have a very high level of accuracy but are subject to limitations imposed by ambient light conditions, physical occlusion, and restrictions related to the equipment. Acoustic systems, which use ultrasound or channel estimation technique, show great promise. They demonstrate comparable accuracy in their work with significantly lower power requirements and hardware complexity. The ability to track eye movements without relying on cameras opens new possibilities for unobtrusive and continuous monitoring in real-world environments.

On the other hand, the development of new algorithms using adversarial and time-frequency approaches makes these systems highly robust and adaptable. They are able to detect even slight differences between specific eye movements and face actions. It should be noted that they are characterized by an ability to operate without much input data. This feature is crucial since personalization is critical for the effective functioning of any human–computer interface. The emergence of hands-free interactive models based on eye movement and acoustic cues may become a significant step in creating more intuitive interfaces.

These developments have important implications for diagnostics and clinical practice. First of all, eye tracking and sound recognition make it possible to obtain objective results about cognitive functioning or auditory skills in those groups of people when traditional procedures cannot be applied. For instance, eye tracking technologies may serve as an alternative to verbal responses in children to detect potential problems with hearing or cognition. The use of a combination of various signs, such as pupil dilation and gaze behavior, may give more information about the level of attention or stress associated with cognitive tasks.

One of the most important benefits of using this methodological approach is the possibility of identifying compensation strategies. People who have hearing problems resort to using other mechanisms to compensate for the deficit. For instance, one such mechanism may be increased visual attention and head movements. Eye tracking gives researchers an opportunity to assess both the success of a compensation strategy and the effects of using assistive devices on the process of communication naturally. The perspective offered by this research helps shift the emphasis from quantitative criteria, like decibel gain, to qualitative aspects such as comfort and usability.

Finally, eye tracking can provide information on the perception of acoustic environments beyond clinical settings as well. The review of studies discussed in this paper shows that the effect of an acoustic environment on visual attention occurs indirectly through emotions and cognition. It has been shown that natural sounds help reduce stress levels and increase attention restoration. In turn, urban noise serves as a form of cognitive load that goes unnoticed when assessing the effects on task performance. However, eye tracking data suggests increased mental effort based on altered patterns of gaze and pupillary response.

**Figure 9:**
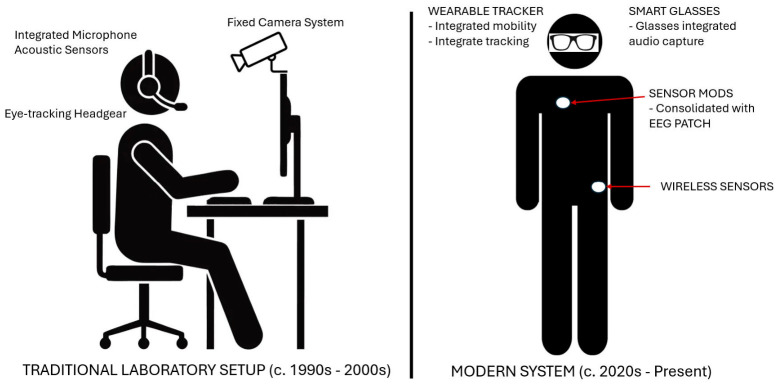
Comparative breakdown transitioning from a constrained, low-sampling traditional laboratory setup (**left**) to a modern, high-ecological-validity framework featuring smart glasses, wireless sensors, and AI analytics (**right**).

To provide historical and technological context, the diagram in Figure 9 contrasts legacy research paradigms with contemporary experimental capabilities in spatial hearing. The left panel depicts the classic experimental envelope, which relied on fixed hardware, restricted participant movement, and desktop processing, heavily limiting ecological validity. Conversely, the right panel highlights the modern transition toward unconstrained, real-world behavioral testing. By shifting to lightweight smart glasses, integrated bio-patches, and cloud-based machine learning pipelines, current systems seamlessly capture natural gaze dynamics in complex acoustic landscapes. This evolution demonstrates how architectural breakthroughs have unlocked highly valid, mobile methodology for auditory cognitive research.

The idea of “hidden cognitive load” has some interesting implications for the planning of workspaces, urban environments, and public environments in general. In other words, it means that not only does noise cause unpleasant sensations but also negatively impacts our basic cognitive functions like attention or decision-making. Thanks to such an effect, eye tracking becomes a valuable technique of evaluation and optimization of our environment’s conditions. The ability of sounds to guide one’s actions, like in choosing particular kinds of food, shows the crucial role of this modality in the perceptual and decision-making process.

However, there is still some space left for research. First of all, inter-individual differences may turn out to be a serious obstacle. Since ocular and auditory systems differ from person to person, we need to create some models and algorithms for continuous adaptation and optimization of parameters. Secondly, environmental noise may significantly reduce the effectiveness of acoustic-based systems due to reverberation and signal interference.

The second crucial aspect to consider is the non-standardization. Due to the variety of experimental procedures, measures of perception, and metrics, it is quite challenging to compare findings among studies. Therefore, developing standardized benchmarks by integrating the visual and auditory aspects of the perception and interaction process would immensely accelerate research efforts. It would be important to take into account not only spatial but also temporal aspects of the process as well as their interaction. Otherwise, the progress will continue to be unstructured and hard to implement in practice.

In the future, there are many possibilities for multimodal systems. Clinical applications may include gaze-based audiological tests that can allow for screening in infants who cannot actively respond to sounds. Human–computer interaction and robotics could benefit from the combination of auditory and visual attention mechanisms to perform human-like behavior. For instance, robots with an “auditory fovea” can adjust their gaze according to the direction of a sound source and respond accordingly.

A third area of opportunity is smart environments. Using eye tracking and acoustic sensors in buildings would allow for continuous optimization of the environmental conditions in real-time, thus alleviating cognitive workload and promoting wellness. Driver monitoring technologies based on eye gaze and auditory signals can help improve road safety and prevent deadly incidents due to driver fatigue or distraction. All of these applications showcase how the synergy between sensing, computation, and artificial intelligence holds potential for revolutionizing our daily lives.

The reviewed literature demonstrates a growing convergence between eye tracking technologies and acoustic analysis within multimodal sensing frameworks aimed at investigating human perception, auditory attention, and behavioral responses. Across the analyzed studies, a recurring trend is the increasing integration of eye tracking measurements with complementary sensing modalities, including physiological monitoring, spatial audio analysis, and machine learning techniques. This evolution reflects the broader transition toward adaptive and human-centered intelligent sensing systems capable of capturing complex perceptual and cognitive processes in dynamic environments.

One of the most significant findings emerging from the reviewed studies concerns the role of eye movements as behavioral indicators associated with auditory perception and attentional allocation. Metrics such as fixation duration, gaze orientation, saccadic activity, blink frequency, and pupil dilation have been widely employed to investigate listening effort, sound localization mechanisms, and cognitive responses to acoustic stimuli. However, the reliability and interpretability of these indicators remain strongly dependent on experimental conditions, environmental complexity, and individual variability.

Several methodological limitations were identified across the reviewed literature. Many studies were conducted under controlled laboratory conditions with relatively small participant samples, potentially limiting the generalizability of the findings to real-world acoustic environments. In addition, substantial heterogeneity exists in terms of sensing configurations, experimental protocols, data acquisition systems, and performance evaluation metrics. This variability complicates direct comparisons among studies and reduces the possibility of establishing standardized benchmarks for eye–acoustic analysis.

Another important challenge concerns the interpretation of multimodal data under noisy or dynamically changing environments. External interference, head movement artifacts, calibration inaccuracies, and differences in participant cognitive states may significantly influence both eye tracking and acoustic measurements. Furthermore, while several studies report positive correlations between gaze behavior and auditory attention, contradictory findings also emerge in complex soundscape scenarios, suggesting that eye movements should not always be interpreted as direct or universal proxies of auditory processing.

The reviewed studies also reveal a growing dependence on machine learning and sensor fusion methodologies for improving the robustness of multimodal perception models. Although these approaches demonstrate promising capabilities in classification accuracy and behavioral prediction, many contributions still lack standardized datasets, transparent validation procedures, and reproducible experimental frameworks. Consequently, future research should prioritize the development of common evaluation protocols, open multimodal datasets, and more ecologically valid experimental scenarios.

Overall, the integration of eye tracking and acoustics represents a rapidly evolving interdisciplinary field with significant potential for applications in human–computer interaction, robotics, healthcare, immersive virtual environments, intelligent monitoring systems, and soundscape assessment. Nevertheless, additional efforts are required to improve methodological consistency, interpretative reliability, and real-world applicability of eye–acoustic sensing systems.

In summary, the combination of eye tracking with acoustic studies presents a paradigm shift that will have wide-ranging effects. As highlighted by the findings reviewed above, integrating information from both visual and auditory streams not only improves system performance and accuracy in location and event detection, but also allows deeper understanding of perceptual and cognitive processes. Although technical problems still need to be addressed, especially regarding generalization of results, the development trajectory is clear. Multimodal systems are becoming increasingly efficient, adaptable, and ecologically valid.

## 6. Conclusions

This literature review systematically examined the integration of eye tracking technologies in acoustic research, focusing on sound localization, auditory event detection, multimodal sensing, and perceptual analysis. The synthesis of the existing body of work shows that eye tracking is increasingly moving beyond a complementary tool and is being incorporated into broader multimodal frameworks aimed at understanding human perception as a unified audiovisual process.

A key contribution of this review is the structured organization of the literature into four main domains—acoustic eye tracking systems, behavioral and physiological monitoring, multimodal data fusion approaches, and application-oriented studies in robotics and environmental systems. This categorization highlights a clear methodological evolution in the field, from isolated experimental paradigms toward integrated sensing frameworks combining visual, auditory, and physiological data. In particular, the reviewed studies consistently indicate that eye-movement features (e.g., fixation behavior, saccadic dynamics, and pupil responses) are widely used as indirect indicators of attention and cognitive processing in acoustic contexts, although their interpretation remains context-dependent rather than universally valid.

From a theoretical perspective, the review supports the view that auditory and visual modalities are tightly interconnected within multisensory perception systems, where eye movements can be considered behavioral correlates of attentional allocation rather than direct proxies of auditory perception. This perspective refines earlier assumptions in the literature and emphasizes the need for more cautious interpretation of eye tracking signals in acoustic environments, especially under complex or ecologically valid conditions.

From a methodological standpoint, the analysis reveals a progressive shift toward the adoption of machine learning techniques, sensor fusion strategies, and real-time multimodal processing pipelines. These advances have improved the capability to handle complex datasets involving heterogeneous signals such as gaze direction, head movement, and spatial audio features. However, the review also highlights persistent fragmentation in experimental designs, measurement protocols, and evaluation strategies, which limits cross-study comparability and the development of standardized benchmarks.

Several important application domains emerge from the literature, including clinical assessment of auditory and cognitive impairments, human–computer interaction, immersive environments, and environmental soundscape analysis. In these contexts, multimodal eye–acoustic systems show strong potential for improving diagnostic accuracy, enhancing user experience, and supporting adaptive system design. Nevertheless, their real-world deployment remains constrained by methodological inconsistencies and limited ecological validation.

It is important to acknowledge the limitations of this review. First, the synthesis is primarily qualitative and does not include quantitative meta-analysis due to the heterogeneity of the available studies. Second, although a structured systematic review methodology was adopted, differences in database coverage and keyword selection may have influenced the scope of the included literature. Third, the absence of standardized effect measures across studies limited the possibility of direct numerical comparison, requiring reliance on thematic and methodological synthesis.

Despite these limitations, this review provides a consolidated and structured overview of a rapidly evolving interdisciplinary field and identifies key gaps that should be addressed in future research. These include the need for standardized experimental protocols, shared multimodal datasets, improved reproducibility of machine learning pipelines, and more rigorous validation in real-world acoustic environments.

## Figures and Tables

**Table 1:** Categorization of the literature sources based on publication types and modes of dissemination, such as journals, conference papers, book chapters, and technical reports, so as to demonstrate the degree of maturity of the research results as well as the main channels for disseminating knowledge in eye tracking and acoustics.

Type	Number of Papers	Main Examples
Scientific Journals	54	Nature Communications, PLoS One, Ear and Hearing, Applied Acoustics
Conference Proceedings	18	IEEE ICRA, CHI Conference, Forum Acusticum, INTER-NOISE
Other (Chapters, Book Series, Reviews)	2	User Centric Studies in Game Translation
Total	74	--

**Table 2:** Most frequent journals and conference venues in the field of eye tracking and acoustic research, highlighting the main academic outlets that serve as reference platforms for the dissemination of multimodal and interdisciplinary studies.

Publishing Channel	Frequency
IEEE (Various Conferences and Journals)	12
ACM (IMWUT/CHI/MUM)	9
Trends in Hearing/Hearing Research	5
Acta Acustica/Applied Acoustics	4
PLoS One/Scientific Reports	3

**Table 3:** Summary of the analyzed studies on audiovisual attention and salience models, reporting for each contribution the authors, sample characteristics, employed technologies, and key findings related to audiovisual salience mechanisms.

Author	Study	Sample	Technologies	Main Results
Marighetto et al. [39]	Audiovisual Toolbox and Dataset	176 eye tracking observers	148 videos (AV/V)	Creation of a dataset and toolbox to analyze the impact of sound on visual salience.
Song et al. [40]	Sound Category Effect	2 groups (AV vs. V)	Eye tracking, Statistical Analysis	Human voices attract the eye more; audio increases the frequency of eye movements.
Xue et al. [41]	Narrative Guidance in VR	N/A	VR, Eye tracking	Spatialized sound guides the eye out of the field of view, especially on the horizontal axis.
Roßkopf et al. [42]	Social Presence in VR	25 participants	VR, Eye tracking	Binaural Audio Plausible audio (HRTF) increases social presence and allows for near-real sound localization.
Roßkopf et al. [43]	Evaluation of binaural auralizations	25 participants	Binaural auralization, head tracking, eye tracking, VR environments, HRTFs	High-quality auralizations achieved near-100% sound externalization and distance perception comparable to real sources. Slight reduction in azimuth accuracy with individual HRTFs.
Król [44]	Children’s facial exploration	49 participants	Eye tracking, pupillometry, audiovisual stimuli	Noise shifts gaze from eyes to mouth to aid speech decoding, reducing socio-emotional cue access. Higher language skills and pupil dilation increase mouth focus, indicating greater cognitive effort and engagement.
Galanda et al. [45]	Webcam-based system	N/A	Eye tracking via webcam, facial landmark tracking, Kalman filter	Gaze orients toward sound sources enabling real-time localization. Head movement compensation improves accuracy. Validated via reaction times; low-cost solution suitable for aviation, noise monitoring, and vehicle control.
Gehmacher et al. [46]	Speech eye tracking	30 participants	Eye tracking, magnetoencephalography (MEG)	Gaze follows attended speech, indicating intelligibility and target selection in multi-speaker settings. Eye movements are linked to neural activity, highlighting their central role in auditory attention and ecological listening.

**Table 4:** Review of the selected studies on the diagnostic uses of eye tracking, featuring for each source the authors, sample features, methods used, and main research outcomes related to eye tracking in acoustics-based tests and diagnosis.

Author	Study	Sample	Technologies	Main Results
Zhang et al. [47]	Early Diagnosis of ASD	108 children (30 ASD, 78 TD)	Eye tracking, Speech Analysis (MFCC), ML (XGBoost)	The multimodal approach achieves 82% accuracy, outperforming single-data models.
Clark et al. [48]	Language Development and ASD	50 children (12–18 months)	Eye tracking, Standardized Assessments	Preference for synchronous speech mediates vocal complexity in males, influencing language.
Van Engen la. [49]	Stress Detection	N/A	Pupillometry, EDA, Neural Networks, Wavelet	Pupil diameter predicts stress with 79.2% accuracy; electrodermal activity is less effective.
Volck et al. [7]	Eye tracking protocol for sound localization assessment	15 participants	Eye tracking, auditory stimuli	High reproducibility and strong gaze–sound correlation. Detected localization error (~5.5°) under simulated hearing loss.

**Table 5:** Summary of the analyzed studies on speech processing and hearing aids, reporting for each contribution the authors, sample characteristics, employed technologies, and key findings related to speech perception, auditory processing, and the role of eye tracking in evaluating assistive hearing systems.

Author	Study	Sample	Technologies	Main Results
Wilroth et al. [50]	AAD Decoding with EEG	Single participant	Mobile EEG, cEEGrid, Eye tracking	Gaze can automatically label the sound source to train speech reconstruction models.
Grimm et al. [51]	Binaural Spatial Filtering	14 participants	Electrooculography, Head-tracking	Gaze-guided filtering improves signal-to-noise ratio (SNR) by up to 7 dB.
He et al. [52]	Neural Tracking (SNR)	14 participants	EEG, Eye tracking (gaze velocity)	Neural tracking depends on the balance between intelligibility and attentional effort (AE).
Eloy et al. [9]	Eye Tracking	64 participants	MEG, Eye tracking	Gaze follows the rhythm of the target speech, demonstrating a shared neural network between vision and hearing.
Lansing et al. [53]	Oculomotor dynamics	17 participants	Eye tracking, lip-reading tasks	Speech triggers gaze shift from eyes to mouth; mouth fixation increases with difficulty; accuracy depends on individual decoding skills more than fixation duration.
Vasilev et al. [54]	Reading under auditory linguistic interference	N/A	Eye tracking, reading tasks, auditory interference	No effect on word recognition; increased regressions and re-reading. Interference is semantic/post-lexical. Eye movements compensate for verbal noise and support comprehension.
Kanerva et al. [55]	Semantic decoding of onomatopoeias in non-native listeners	N/A	Eye tracking (visual world paradigm), free association task	Iconic cues improve recognition accuracy. Response clusters reveal guiding or misleading acoustic patterns.

**Table 6:** Summary of the analyzed studies on attention classification and professional skill assessment, reporting for each contribution the authors, sample characteristics, employed technologies, and key findings related to the use of eye tracking for evaluating attentional patterns and expertise levels in task-specific environments.

Author	Study	Sample	Technologies	Main Results
Abdelrahman et al. [56]	Classification of 4 Types of Attention	22 participants	Thermal imaging, Eye tracking	Classification of sustained, alternating, selective, and divided attention with AUCs up to 87%.
Ito et al. [57]	Empathic Communication	2 nursing students	Eye tracking, Speech analysis, Video	Protocol that visualizes empathy through gaze direction (94% on the face) and changes in sound pressure.
Hartnett et al. [58]	Advertising Attention	261 participants	Eye tracking, HR, EDA, EEG	Heart rate outperforms eye tracking in distinguishing levels of attention to video advertisements.
Lehtilä et al. [59]	Multilingual Fluency (L1/L2/L3)	6 multilingual speakers	Eye tracking, Qualitative speech analysis	Gaze reveals cognitive effort and stalling mechanisms during production in different languages.
Doyle et al. [8]	Auditory cues facilitate visual target detection	10 participants	Eye tracking, audiovisual task paradigm	Synchronized sounds speed visual detection without accuracy loss; effect independent of spatial congruence.
Gerdes et al. [60]	Cross-modal effects of auditory emotion on visual attention	48 participants	Eye tracking, free-viewing paradigm, audiovisual stimuli	Emotional valence and spatial congruence guide gaze. Negative sounds speed orienting; congruent audiovisual emotion enhances sustained attention and attentional prioritization.
Yang et al. [61]	Gaze-driven audio for immersive museum experience	14 participants	Eye tracking, spatial audio, interactive museum system	Gaze-contingent audio increases attention to areas of interest; improves concentration and aesthetic engagement; enables active multisensory exploration of paintings.
Baumann et al. [62]	Neural mechanisms of eye pursuit and auditory attention conflict	19 participants	Eye tracking, smooth pursuit eye movements (SPEM)	Divergent gaze and auditory attention increase parietal/frontal activation; strong neural resource recruitment for coordinating oculomotor and auditory processing.

**Table 7:** Summary of the analyzed studies on methodological contributions and hardware/software innovations for the processing of ocular and acoustic data, reporting for each contribution the authors, sample characteristics, employed technologies, and key advances in data acquisition, synchronization, and multimodal signal processing.

Author	Study	Sample	Technologies	Main Results
Nurlatifa et al. [10]	Engineering/Review	N/A	Video-based systems	Definition of an event detection taxonomy and noise reduction standards.
Simpsi et al. [11]	Wearable Technology	N/A (Dataset focus)	Event-based sensors	Development of an annotation pipeline for energy-efficient smart glasses.
Qiao et al. [63]	Computer Vision	AI Multi-face dataset	(AVM-Net), Audio–visual tracking	Integration of audio and physiognomic signals improves visual saliency prediction.
Sidaty et al. [64]	Videoconferencing	Users in digital landscape	Multimodal saliency maps	Real-time speaker localization optimizes gaze prediction models.

**Table 8:** Summary of the analyzed studies on the correlations between brain activity (EEG/MEG), vestibular function, and oculomotor behavior in response to auditory stimuli, reporting for each contribution the authors, sample characteristics, employed technologies, and key findings on cross-modal neural and sensory integration.

Author	Study	Sample	Technologies	Main Results
Pomper et al. [12]	Cognitive Psychology	Healthy Adults	EEG + Eye tracking	Eye–ear misalignment increases the central theta band (cognitive effort).
Wang et al. [65]	Sleep Neuroscience	Awake/Asleep Subjects	EEG + MEG	NREM sleep preserves basic hearing but suppresses cortical spatial localization.
Sarac et al. [66]	Otolaryngology	Subjects with VNG Testing	Videonystagmography (VNG)	Auditory cognitive tasks (“what/where”) degrade reflex oculomotor parameters.
Van Barneveld et al. [67]	Vestibular Perception	Healthy Adults	Sinusoidal Rotation + Headphones	The audiogyral illusion is mediated by vestibular-induced eye position.
Zhang et al. [68]	Cognitive Ergonomics	24 Operators	Eye tracking + Geo-surveillance	Spatialized audio reduces visual overload in complex monitoring interfaces.

**Table 9:** Summary of the reviewed research on pediatric and clinical subjects that is intended to validate hearing assistance equipment and investigate motor-sensory development, providing for each study information about the authors, the participants, used technology, and results relevant to hearing assistance effectiveness and developmental assessment.

Author	Study	Sample	Technologies	Main Results
Alemu et al. [69]	Psychoacoustics	33 Children, 17 Adults	Eye-tracker + Gyroscopes	Gaze precedes the head in sound pointing; occlusion reverses motor patterns.
Coudert et al. [70]	BCI Rehabilitation	18 Children (BCI vs. NH)	VR + Motion Tracking	Head movements are a vital strategy to compensate for the limitations of cochlear implants.
Eklöf et al. [71]	Pediatric Audiology	22 Children (0.5–5 years)	Saccadic Reflexes	Localization Latency (SLL) is a reliable marker of auditory maturation.
Eklöf et al. [72]	Clinical Diagnostics	8 Adults (simulated SUHL)	Eye tracking (reflex)	Unilateral hearing loss increases SLL proportionally to the deficit.
Asp et al. [73]	Prosthetics	11 Subjects (CHL)	Bone Conduction Implants	BCIs significantly improve the error rate (EI) assessed via eye tracking.
Eklöf et al. [74]	Signal Coding	30 Children (BCI)	Fine Structure (FS)	Strategies The FS strategy allows for the detection of ITD, but localization remains intensity driven (ILD).

**Table 10:** Summary of the analyzed studies conducted in virtual reality and gaming environments on multisensory dynamics of sound localization, reporting for each contribution the authors, sample characteristics, employed technologies, and key findings related to audiovisual integration and spatial auditory perception in immersive contexts.

Author	Study	Sample	Technologies	Main Results
Kudla et al. [75]	Audiovisual Translation	Video Game Players	Game Analytics + Tracking	Digital media interactivity alters gaze patterns compared to passive viewing.
Valzolgher et al. [76]	Multisensory Perception	36 Subjects (NH)	VR + Cinematic Tracking	A minimal visual frame accelerates gaze reaction towards the sound source.
Fischer et al. [77]	Audiological Methodology	12 Subjects (NH)	Dynamic Tests + Touchpad	Freedom of head movement optimizes dynamic stimulus tracking.

**Table 11:** Summary of studies on integrated eye tracking systems based on acoustic signals and physiological monitoring, highlighting the technologies used, multimodal fusion methods, parameters analyzed, and main application areas.

Author	Study	Sample	Technologies	Main Results
Li et al. [13]	GazeTrak	20 participants	Acoustic-based eye tracking with loudspeakers and four microphones; deep learning model	3.6° accuracy within sessions and 4.9° across sessions; 83.3 Hz refresh rate; low power consumption with high performance
Sun et al. [78]	PMUT Eye Tracking	N/A	Ultrasound-based eye tracking using microelectromechanical transducer arrays (PMUT); time-of-flight estimation	Real-time tracking of eye direction and rotation with high precision; lightweight and suitable for portable applications
Liang et al. [79]	Blink Monitoring System	N/A	Ultrasound-based sensing with piezoelectric MEMS transducer; unsupervised machine learning	Reliable real-time blink detection; non-invasive; compatible with standard manufacturing; suitable for continuous monitoring
Lu et al. [80]	Ultrasound Simulation Platform	N/A	Synthetic face/eye model; ultrasound simulation; machine learning algorithms	Mean squared error of 0.085° (no displacement) and 0.756° (with displacement); useful for optimizing eye tracking system design

**Table 12:** Summary of research on acoustic sensing in wearable devices for facial interaction and behavioral analysis.

Author	Study	Sample	Technologies	Main Results
Sun et al. [81]	EyeGesener	16 participants	Acoustic sensing with speakers and microphones; channel estimation; machine learning with adversarial training	Recognizes 8 eye gestures; F1 score of 0.93; false alarm rate of 0.03; high usability in real-time interaction
Xie et al. [82]	Facial Action Recognition	26 participants	Acoustic sensing; time-frequency analysis; deep learning models	Identifies 6 facial actions with F1 score of 0.92; effective for hands-free interaction
Mahmud et al. [83]	MunchSonic	12 participants	Ultrasonic acoustic sensing; deep learning models	F1 score of 0.935; detects eating actions and intake frequency; 2-s temporal resolution
Li et al. [84]	SonicID	40 participants	Ultrasonic facial scanning; biometric feature extraction; deep learning (binary classification)	97.4% true positive rate; 4.3% false positive rate; 96.6% balanced accuracy; stable across sessions
Li et al. [85]	EyeEcho	22 participants (12 controlled + 10 natural)	Acoustic sensing with speakers and microphones; machine learning models	High accuracy with only 4 min of training; consistent real-time performance in everyday scenarios

**Table 13:** Overview of studies on multimodal systems integrating eye tracking and acoustic information, highlighting sensing modalities, data fusion strategies, extracted features, and key application areas.

Author	Study	Sample	Technologies	Main Results
Kotus et al. [86]	Camera Guidance System	5 participants (PhD students)	Eye tracking; vector acoustic sensors; image processing and camera control algorithms	Improved detection and tracking of relevant events; enhanced user interaction through multimodal control
Asp et al. [87]	Sound Localisation Assessment	20 participants (12 children + 8 adults)	Eye tracking with corneal reflection; auditory and visual stimuli; Smart Eye Pro system	Fast and objective measurement; high accuracy in adults; lower but improving accuracy in children; potential clinical application
Ohneiser et al. [88]	Air Traffic Voice System	2 participants	Eye tracking; mouse interaction data; visual attention analysis	Increased prediction accuracy; reduced errors; visual confirmation can replace manual input, improving efficiency
Langner et al. [89]	Driver Attention Monitoring	N/A	Eye tracking; computer vision; head tracking; human–machine interface with visual and acoustic alerts	Detects driver inattention; provides timely warnings; improves road safety
Seong et al. [90]	Badminton Performance Dataset	25 participants	Eye tracking; motion capture; muscle signals; foot pressure sensors; video recording	Enables biomechanical analysis; supports machine learning for personalized training; large annotated dataset (7763 movements)

**Table 14:** Overview of studies on robotic audition and audio–visual integration systems, highlighting sensing architectures, multimodal fusion strategies, processing methods, and key applications in robotic perception and human–robot interaction.

Author	Study	Sample	Technologies	Main Results
Trifa et al. [91]	Human–Robot Interaction System	N/A	Multisensory integration (acoustic and visual data); sound localization algorithms; distributed control framework; speech recognition and visual tracking	More natural and effective robot responses; improved sound source localization; enhanced human–robot interaction
Nakadai et al. [92]	Active Robotic Hearing	N/A	Audio–visual fusion; adaptive directional filtering; multiple acoustic models; motion-based active audition	Improved localization, signal separation, and speech recognition in noisy environments; effectiveness of active listening through motion (“auditory fovea”)

**Table 15:** Overview of studies on soundscapes and psychological restoration in natural and urban environments, highlighting experimental designs, perceptual and physiological measures, acoustic indicators, and main findings related to stress recovery and attention restoration.

Author	Study	Sample	Technologies	Main Results
Li et al. [15]	Tourist Attention & Emotion	N/A	Eye tracking; audiovisual and textual stimuli applied to natural and urban images	Auditory stimuli increase attention to natural elements and positive emotions; text directs attention to built elements; natural scenes linked to better emotional response, especially under low stress
Fei et al. [96]	School Soundscapes	101 participants (schoolchildren)	Eye tracking; virtual audiovisual scenes; physiological measures (electrodermal activity, heart rate)	Natural sounds improve perception and cognitive recovery; birdsong most effective; traffic increases fatigue; effects depend on spatial context
Liang et al. [16]	Campus Soundscapes	30 participants	EEG; eye tracking; acoustic measurements; questionnaires	Natural sounds improve emotions and recovery; artificial noise causes negative effects; acoustic comfort correlates with positive emotional states
Kou et al. [97]	Multisensory Nature Recovery	405 participants	Eye tracking; questionnaires; interviews; acoustic and location data	Visual and other sensory stimuli directly support attention recovery; soundscape acts indirectly via emotion; stronger effects with higher nature exposure reduction
Zhu et al. [98]	Bamboo Forest Audio-Visual Study	32 participants	Eye tracking; audiovisual experimental stimuli	Natural sounds improve perception and reduce fatigue; recreational/ornamental spaces more restorative; sound guides visual attention toward natural elements
Ren et al. [99]	Rural Landscape Perception	20 participants	Eye tracking; audiovisual stimuli in lab settings	Natural/musical sounds increase preference and tranquility; sound significantly alters visual attention to landscape elements
Weng et al. [100]	Forest Bathing	41 participants	Eye tracking; physiological measures (heart rate variability, skin conductance); questionnaires	Natural and cultural stimuli reduce stress, improve physiological regulation and emotional state; overall improvement in well-being

**Table 16:** Overview of studies on the influence of soundscapes on visual attention and multisensory perception, highlighting experimental setups, auditory conditions, eye tracking metrics, and key findings on cross-modal interactions between hearing and vision.

Author	Study	Sample	Technologies	Main Results
Oberman et al. [101]	Visual–Soundscape Interaction	N/A	Eye tracking; questionnaires; 360° VR videos; spatial audio recordings	Visual information significantly alters soundscape perception; differences between vision- and sound-driven perception; context-dependent and multi-factorial nature of soundscape experience
Hirway et al. [102]	Immersive Audio Attention	73 participants	Eye tracking; head-orientation tracking; physiological measures (heart rate, pupil dilation); 360° immersive video	Spatial audio increases immersion and improves visual attention guidance; potential for bandwidth-efficient immersive content design
Wright et al. [103]	Multisensory Localization	48 participants	Eye tracking; audiovisual sensory substitution; laboratory-based visual scenes	Visual context improves auditory target localization; eye movements align with auditory targets even when responses differ; strong multisensory integration effects
Kothinti et al. [104]	Auditory Salience Study	275 participants; one scene pair repeated every ~18 subjects on average.	Online platform; dichotic listening task; eye tracking; crowdsourcing validation	Auditory salience depends on complex sound characteristics beyond low-level acoustic features; natural sound richness better captured with large-scale behavioral data

**Table 17:** Overview of studies on soundscape, cognitive load, and human behavior, highlighting experimental conditions, acoustic environments, cognitive and behavioral metrics, and key findings on mental workload, attention, and decision-making processes.

Author	Study	Sample	Technologies	Main Results
Grenzebach et al. [105]	Noise and Cognitive Load	N/A	Eye tracking; pupillometry; EEG/brain activity; cardiovascular and endocrinological measures; behavioral markers	Irrelevant noise increases cognitive load and mental effort even without affecting observable performance; highlights need to address hidden cognitive strain in workplaces
Peng-Li et al. [106]	Music & Food Choice	215 participants (114 China, 101 Denmark)	Eye tracking; behavioral food-choice task; personalized music stimuli	‘Healthy’ music increases healthy food selection and visual attention to healthy options; eye movements partially mediate the effect of sound on decision-making

## Data Availability

Not applicable.
